# Structural mechanisms underlying distinct binding and activities of 18:0 and 18:1 lysophosphatidic acids at LPA1 receptor

**DOI:** 10.1371/journal.pcbi.1013825

**Published:** 2026-04-24

**Authors:** Ayobami Diyaolu, Peter Obi, Pravita Balijepalli, Kathryn E. Meier, Senthil Natesan

**Affiliations:** College of Pharmacy and Pharmaceutical Sciences, Washington State University, Spokane, Washington, United States of America; Changchun Institute of Applied Chemistry Chinese Academy of Sciences: Chang Chun Institute of Applied Chemistry Chinese Academy of Sciences, CHINA

## Abstract

Lysophosphatidic acids (LPAs) are bioactive lipids that regulate numerous physiological functions in humans. Cell signaling by LPAs is mediated mainly via six LPA receptors (LPA1-6), class A G protein-coupled receptors (GPCRs). Among these, LPA1 is recognized to play an essential role in cell proliferation, survival, migration, and tumorigenesis. Despite the structural similarity, 18:0-LPA and 18:1-LPA exhibit distinct functional responses in cell lines overexpressing LPA1. Specifically, our in vitro studies show that 18:1-LPA induces greater Erk activation than 18:0-LPA in PC-3 human prostate cancer cells. The structural basis underlying this differential receptor activation has not been previously studied. Using classical molecular dynamics and enhanced sampling techniques, we examined the access and binding mechanisms of the two LPA species to the active state LPA1 receptor. The results show that 18:0-LPA and 18:1-LPA adopt distinct and dynamic poses in the orthosteric pocket despite their similar starting configurations. Mainly, the alkyl chains of the ligands exhibit distinct orientations and residue interactions, leading to differential conformational changes in key activation switches on the conserved CWxP and PIF structural motifs of the receptor. Also, there are significant differences in interhelical interactions at the intracellular end of the transmembrane helices 1, 3, 6, and 7. These distinct arrangements lead to striking differences in LPA1 interactions with the Gα-helix of the heterotrimeric Gi-protein. Notably, 18:0-LPA and 18:1-LPA exhibit similar membrane partitioning characteristics and receptor entry processes through aqueous paths. Our comprehensive in-silico studies offer valuable structural insights into the observed differences in functional responses by 18:0- and 18:1-LPA.

## Author summary

Cells communicate partly through lipid-derived signaling molecules, and lysophosphatidic acids (LPAs) are among the most biologically active of these. They act on LPA receptors, a subfamily of G protein-coupled receptors, to regulate processes like cell proliferation, migration, and survival, with LPA1 playing a particularly prominent role in cancer contexts. LPA species differ from one another primarily in their alkyl chains, and while these differences look minor, they can meaningfully change how well a ligand activates its receptor. We examined this question for two 18-carbon LPAs: 18:1-LPA has a cis double bond at C9-C10, while 18:0-LPA is fully saturated. Our cell experiments show that 18:1-LPA activates the Erk signaling pathway more effectively than 18:0-LPA in LPA1-expressing prostate cancer cells. To understand why, we ran extensive atomistic simulations of both ligands bound to LPA1. The results show that the double bond subtly constrains the shape the alkyl chain adopts in the orthosteric pocket, and this geometric difference is enough to shift the balance of conserved receptor activation switches and alter how productively the receptor engages the Gi protein. Membrane partitioning and receptor access are similar for both ligands, so the efficacy difference is rooted in what happens after binding.

## Introduction

Lysophosphatidic acid (LPA) receptors belong to a subfamily of class A G protein-coupled receptors (GPCRs) known as Lipid GPCRs. Lipid GPCRs also include sphingosine 1-phosphate receptors and cannabinoid receptors and are named based on the lipid-derived origins of their endogenous agonists [1–3]. The LPA receptor family includes six receptors (LPA 1–6), among which LPA1 is the most studied [4,5]. LPA1 is widely distributed in human tissues, and its activation promotes cell proliferation, survival, migration, and tumorigenesis [6–8]. Notably, LPA1 has been implicated in cancer cell survival and nourishment of the tumor microenvironment [7]. LPA1 is promiscuous regarding G-protein coupling [9]. It can couple to the G_12/13_ protein to activate phospholipase C, which leads to calcium mobilization [6,10]. It can also bind to Gi/o protein, through which it modulates the RAS pathway, ultimately leading to the phosphorylation of the extracellular signal-regulated kinase (Erk) [11].

LPA1 is activated by endogenous lysophospholipids known as lysophosphatidic acids (LPAs). LPAs are nearly ubiquitous in human body tissues and fluids [8,12]. Structurally, they possess a phosphate head, glycerol backbone, and an alkyl chain that varies in length and degree of unsaturation, giving rise to numerous LPA species [13,14]. The most abundant LPA species in the human body include 14:0, 17:0, 18:0, 18:1, and 20:4 LPAs [15]. The relative activities of various forms of LPA have been reported for a handful of cellular models in which the relevant LPA receptor was defined. In 2000, Bandoh and colleagues examined the activities of an extensive series of LPA analogs on calcium mobilization in Sf9 insect cells transfected with LPA1, LPA2, or LPA3 receptors [16]. They reported similar potencies for 18:1-, 18:2-, 18:3-, and 20:4-LPAs in cells expressing LPA1 receptor. There was negligible agonist activity for 12:0- and 14:0-LPAs and lower potency for 16:0- and 18:0-LPAs than for 18:1-LPA. Subsequently, another study examined the effects of multiple LPA species on calcium mobilization and Erk activation in human lung fibroblasts, where LPA1 is the predominant endogenous LPA receptor [11]. With respect to Erk activation, the study reported that 20:0-, 20:4-, 18:2-, 18:3-, 17:0–16:0-, and 14:0-LPAs had lower potency than 18:1-LPA. Also, longer chain LPAs (18:1 and 20:4) have shown greater activity than shorter chain LPAs (14:0 and 16:0) [11,15]. This study also reported possible bias between calcium mobilization and Erk activation for some ligands (16:0, 17:0, and 18:2), suggesting that relative efficacy can depend on the measured response.

Our group has characterized LPA receptors and responses in prostate cancer cell lines, in which LPA stimulates cell proliferation. We previously showed that 18:1-LPA was most effective at increasing Erk activation in Du145 prostate cancer cells, 18:0-LPA acted as a partial agonist, and 16:0-, 14:0-, and 6:0-LPAs elicited little or no response [17]. Subsequent work by our group confirmed that LPA1 is the receptor responsible for 18:1-LPA-induced proliferation and migration in Du145 and PC-3 human prostate cancer cells [18]. In subsequent work, we have used PC-3 as a well-characterized cell line in which to explore the cellular responses mediated by LPA via the LPA1 receptor. In summary, previous findings indicate that 18:1-LPA is the most potent and efficacious agonist for the LPA1 receptor. Shorter saturated LPA species and other 18-carbon LPAs with additional saturation/unsaturation have varying efficacies in different model systems but are generally less active than 18:1-LPA. Data interpretation can be complicated by the fact that most mammalian cells express multiple LPA receptors since the relative activities of different LPA species differ between LPA receptors [4,16,19]. However, PC-3 cells serve as a reliable model for responses mediated by LPA1 [20].

**Fig 1:**
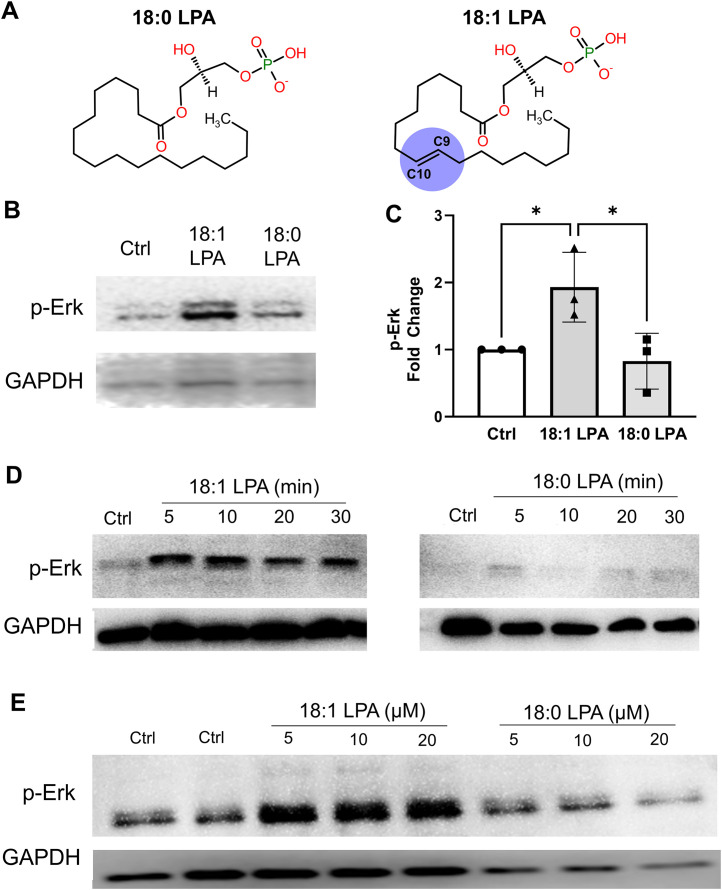
Effects of 18:1- and 18:0-LPA on Erk activation in PC-3 human prostate cancer cells. **A)** 2D structures of 18:0-LPA and 18:1-LPA. Both molecules are structurally identical, with a polar glycerol-phosphate group at one end and an 18-carbon alkyl chain at the other. The only difference is the double bond between atoms C9 and C10 in 18:1-LPA, which is absent in 18:0-LPA. To aid visual discrimination of the unsaturation, the C9 = C10 double bond in 18:1-LPA is indicated by a light purple circular highlight. **B)** Serum-starved PC-3 cells were incubated with and without (“Ctrl”) 10 µM 18:1-LPA and 18:0-LPA for 15 minutes. Whole-cell extracts were immunoblotted for activated phospho-Erk (p-Erk) and for GAPDH (loading control). **C)** Erk phosphorylation was quantified from experiments in which serum-starved PC-3 cells were incubated with and without 10 µM 18:1-LPA and 18:0-LPA for 10 minutes. The p-Erk signal was quantified by densitometry, utilizing background subtraction and normalization to GAPDH. The p-Erk levels in treated cells were divided by levels in untreated control cells within each experiment. Each data point represents the mean ± **S.**D. of values from four separate experiments. Statistical significance was determined using the Student’s t-test. **D)** Serum-starved PC-3 cells were incubated with 10 µM 18:1-LPA or 18:0-LPA for 5-30 minutes. Whole-cell extracts were immunoblotted for p-Erk and GAPDH. All lanes are from the same experiment, gel, and blot, except that p-Erk and GAPDH were analyzed from separate gels using the same samples. **E)** Serum-starved cells were incubated for 10 minutes with the indicated concentrations of 18:1- or 18:0-LPA; the control (“Ctrl”) was untreated. Whole-cell extracts were immunoblotted for p-Erk and GAPDH. Previous studies from our lab have established that LPA treatment for up to 15 minutes does not alter total Erk levels in PC-3 cells [17,41].

18:1-LPA is nearly identical to 18:0-LPA in structure and almost every physicochemical property; the only difference is the presence of a double bond between C9 and C10 carbons in 18:1-LPA, whereas 18:0-LPA is fully saturated (Fig 1A and Table A in S1 Appendix) [21]. Both molecules are amphiphilic, with 18:0-LPA (Clog *P* = 5.85) slightly more lipophilic than 18:1-LPA (Clog *P* = 5.49) [22]. The two LPAs are highly flexible due to their many rotatable bonds (18:1-LPA = 21, 18:0-LPA = 22) and thus can potentially exist in many conformations under physiological conditions. Despite their similarities, 18:1-LPA is a more efficacious agonist for LPA1 than 18:0-LPA, as introduced earlier. The difference in the saturation of the alkyl chain may give rise to the differential behavior of the two species in lipid bilayers and in their interactions with the membrane-embedded receptors. In general, saturation in fatty alkyl chains of phospholipids has been shown to impact the biophysical characteristics of the membrane, including fluidity, bilayer thickness, curvature, and stability, which could eventually affect important cellular and biological functions [23,24]. Although the role of fatty alkyl chain length on the activity of LPAs on lipid GPCRs has been studied before [25], to our knowledge, no studies have examined the mechanistic basis for the impact of saturation-unsaturation on the activities of LPA species at LPA1.

Recent developments in structural biology (cryo-EM and X-ray crystallography) led to the elucidation of the inactive LPA1 receptor bound to antagonists in 2015 [26]. However, it wasn’t until recently that the active structure of LPA1 bound to endogenous 18:1-LPA was elucidated [27]. Subsequent developments in the field led to several active LPA1 receptor structures with co-crystallized lipid and non-lipid agonists [28,29]. Compared to the inactive receptor, several structural rearrangements occur in the orthosteric pocket of active LPA1 involving several hydrophobic residues, including L132^3.36^, W210^5.43^, F267^6.44^, W271^6.48^, L297^7.39^, and A300^7.42^ [26]. LPA1 receptor activation involves a large outward movement of the intracellular end of transmembrane helix 6 (TM6) and an inward movement of TM7 toward TM3 [28]. These changes lead to the opening of LPA1’s intracellular cavity for coupling to the Gα- helix of the Gi protein. Similar to other Class A GPCRs [30–33], signal transduction between the binding site and G-protein coupling interface could involve conserved structural domains, including the CWxP motif (C^6.47^, W^6.48^, x^6.49^, and P^6.50^), PIF motif (P^5.50^, I^3.40^, and F^6.44^), and NPxxY motif (N^7.49^, P^7.50^, x^7.51^, x^7.52^, and Y^7.53^). However, most of these structural details on LPA1 activation by agonists have been based primarily on the static structures from cryo-EM studies. There is limited information on comparative changes in the conformational dynamics of active state LPA1 in the presence of endogenous agonists, which can be investigated via molecular dynamics (MD) simulations.

In addition, the mechanistic details of how 18:0-LPA and 18:1-LPA access the orthosteric binding site of LPA1 remain poorly understood. Although many of the endogenous ligands enter their orthosteric sites in GPCRs from the extracellular aqueous phase, for multiple receptors, ligand entry through transmembrane helices from the surrounding membrane lipids has been reported [34–38]. The mode of entry of LPA species could play a role in “pre-organizing” ligands into orientations and conformations that would affect the binding kinetics and interactions within the binding site [13,39]. The polar head groups and hydrophobic alkyl chains of the LPA species should exhibit distinct affinities for the aqueous phase and various polar and nonpolar functional groups of the membrane lipids. These differences could affect their partitioning characteristics, such as preferred bilayer depth, orientation, and conformations. The membrane partitioning characteristics of these ligands are yet to be examined and correlated with their access and binding mechanisms to the orthosteric site of LPA1.

In this study, we first investigated the efficacy of 18:0-LPA and 18:1-LPA in activating Erk in PC-3 human prostate cancer cells. LPA1 is the endogenous receptor predominantly responsible for LPA-induced proliferation in this cell line [40]. Next, to gain structural insights into the activation process, we performed unbiased atomistic simulations of both 18:0 and 18:1-LPA bound to LPA1 in the presence and absence of the heterotrimeric Gi protein. Subsequently, we elucidated the plausible access paths of the ligands to the orthosteric site of the LPA1 receptor using well-tempered metadynamics (WT-metaD). Also, we utilized steered MD and umbrella sampling techniques to elucidate the membrane partitioning characteristics of both LPA species to determine their energetically favorable bilayer locations, orientations, and conformations within a model membrane bilayer. In addition, we computed the relative binding free energies of 18:0-LPA and 18:1-LPA to LPA1, including the contribution of individual binding site residues using the molecular mechanics Poisson-Boltzmann surface area (MM/PBSA) technique. Comprehensive analyses of both classical and enhanced sampling simulations revealed distinct and dynamic binding orientations and critical residue interactions of the two LPA species within the orthosteric site. The similarities and differences in hydrophobic interactions, rotameric shifts, several activation signatures commonly observed in other GPCRs, and residue-residue communication between the binding site residues and G protein coupling interface residues were analyzed to gain valuable insights into the differential activities of the two LPA species.

## Results

### 18:1-LPA is more efficacious than 18:0-LPA at activating Erk in PC-3 human prostate cells

PC-3 human prostate cells respond to 18:1-LPA by increasing proliferation and migration; these responses are mediated by the LPA1 receptor [40]. Erk activation, which is involved in mitogenesis, is an early response to 18:1-LPA in PC-3 cells [17,41]. We, therefore, used LPA-induced Erk activation to compare the relative activities of 18:1- and 18:0-LPA in these cells. As shown in Fig 1B, 18:1-LPA activated Erk (as assessed by an antibody recognizing phosphorylated Erk) to a greater extent than 18:0-LPA when both ligands were applied at a concentration of 10 µM for 15 minutes. This response was quantified by densitometry from multiple experiments (Fig 1C). The results show that Erk phosphorylation in response to 18:1-LPA was statistically different from untreated cells and 18:0-LPA-treated cells; 18:0-LPA did not significantly increase Erk phosphorylation. These differences in efficacy between 18:1- and 18:0-LPA are consistent with those published previously for Du145, another human prostate cancer cell line [17], except that 18:0 was a partial agonist in Du145 cells. The relative efficacy is also consistent with the induction of cellular communication network (CNN) proteins, an alternative and more delayed LPA response observed in our study [42]. A time course experiment was carried out to test whether the different efficacies of 18:1- and 18:0-LPA reflected differences in the kinetics of the response (Fig 1D). The results show that 18:0-LPA did not elicit substantial Erk activation at times from 5-30 minutes, as compared with the response to 18:1-LPA, which was maximal at 10 minutes but prominent at all time points tested. Next, the effects of different concentrations of 18:1- and 18:0-LPA were compared to determine whether 18:0 might be less potent than 18:1-LPA (Fig 1E). The results indicate that the response to 18:0-LPA was negligible at all concentrations tested (5–20 µM), while 18:1-LPA elicited Erk activation throughout this dose range. In summary, the signal transduction studies, which were conducted at early times after LPA addition, indicate that 18:1-LPA is an agonist for PC-3 cells expressing endogenous LPA1, while 18:0-LPA has negligible efficacy.

### Binding modes and residue interactions of LPAs in the LPA1 orthosteric site

**Fig 2:**
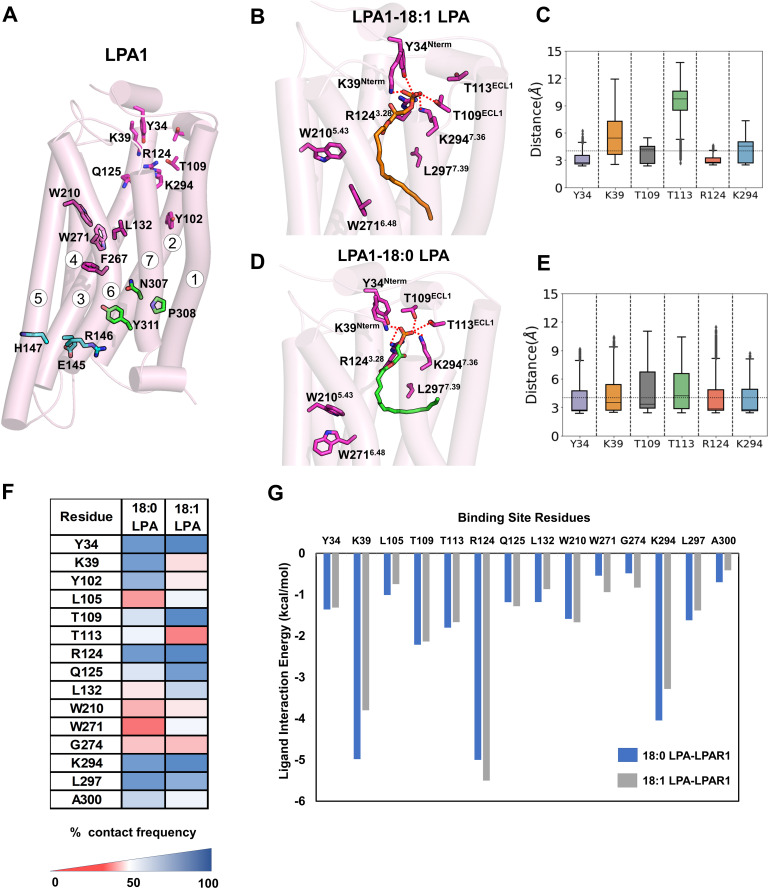
Binding poses, polar contacts, contact fingerprints, and per-residue interaction energies 18:1-LPA and 18:0-LPA in LPA1 (from unbiased MD simulations). **(A)** Secondary structure representation of LPA1 in the active state (PDB ID 7TD1) with seven transmembrane helices labeled 1-7. Sidechains of residues implicated in access and binding at the upper vestibule, orthosteric recognition/signal transmission, and activation switches are highlighted (light magenta, dark magenta, green, and cyan, respectively). **(B)** Most preferred binding pose of 18:1-LPA (orange licorice) in the orthosteric pocket was obtained from 500 ns unbiased MD. Key polar contacts to Y34^Nterm^, K39^Nterm^, T109^ECL1^, R124^3.28^, and K294^7.36^ are indicated (dotted red lines). **(C)** Stability of 18:1-LPA polar interactions shown as distance boxplots (median, quartiles; whiskers to full range) for the residue pairs in panel **B.** All distances are measured between the nearest heavy atom of the ligand’s glycerophosphate group and the nearest heavy atom of the indicated residue. **(D)** Most preferred binding pose of 18:0-LPA (green licorice) in the orthosteric pocket from 500 ns unbiased MD, with corresponding polar contacts highlighted. **(E)** Distance boxplots for 18:0-LPA polar interactions (same residue set and distance definition as in panel **C)**. **(F)** Contact-frequency heatmap (cutoff 4 Å) for ligand-residue contacts across binding-site residues, comparing 18:0-LPA and 18:1-LPA. Values report the percentage of simulation frames in which a residue lies within 4 Å of the ligand. **(G)** Per-residue interaction energies (MM/PBSA components summed; negative values favor binding) for key binding-site residues with 18:0-LPA (blue) and 18:1-LPA (gray). Definition and validation of “most-preferred binding pose:” bound-state conformations from three 500 ns replicates were clustered by ligand heavy-atom RMSD; the largest-occupancy cluster was selected and its medoid is shown in 2B (18:1-LPA) and 2D (18:0-LPA). The displayed poses were cross-validated against the contact-frequency heatmap (2F, heavy-atom cutoff 4 Å): the pose’s ligand-residue contacts reproduce the high-occupancy contacts observed over the trajectories. All metrics in panels C-G aggregate all three 500-ns replicates per ligand.

To assess the differential ligand binding and conformational dynamics of LPA1 upon binding to the two LPAs, we utilized a recently published cryo-EM structure of LPA1-Gi protein complex bound to 18:1-LPA (PDB ID: 7TD1). The receptor is illustrated in its secondary structure representation with critical orthosteric binding site residues and residues involved in signal transduction and activation processes (Fig 2A). In the static structure, the alkyl chain end of 18:1-LPA adopts a nearly U-shaped orientation within the pouch-shaped pocket created by steric restraints from W210^5.43^ at the base of the binding site [27] (Fig AA in S1 Appendix). The ligand’s alkyl chain is also in contact with residues D129^3.33^, L132^3.36^, W271^6.48^, and L297^7.39^, lining the pocket and is oriented towards the space between TM1 and TM7. Since no experimental structure of LPA1 with 18:0-LPA is available, we performed several rounds of molecular docking to obtain the plausible binding poses. Ligand poses with the best docking scores (ranging from -12 to -15 kcal/mol) had the phosphate headgroup oriented towards the N-terminal interacting with residues Y34^Nterm^ and K39^Nterm^, similar to the 18:1-LPA bound structure. At the same time, the alkyl chain forms numerous hydrophobic interactions with residues lining the base of the orthosteric pocket (Fig AB in S1 Appendix). Overall, the selected pose of 18:0-LPA is within 4 Å of several binding site residues, including T109^ECL1^, T113^ECL1^, R124^3.28^, Q125^3.29^, D129^3.33^, W210^5.43^, W271^6.48^, E293^7.35^, and K294^7.36^, as previously described for 18:1-LPA^28^.

To investigate the dynamics of the ligands within the receptor site, we performed classical MD simulations for 500 ns (three replicates) of the LPA1-ligand receptor complexes in the presence and absence of the heterotrimeric Gi-protein, totaling three microsecond-long simulations per ligand. The longevity of polar and nonpolar residue-ligand interactions was quantified as contact frequency and presented as a heatmap (Fig 2F). The contact frequency represents the fraction of the simulation time during which a given binding site residue is within 4 Å of the ligand atoms. All contact and energy analyses aggregate data across all three replicates per ligand.

The MD simulations reveal significant conservation of polar contacts for both LPA species. The negatively charged phosphate group of 18:1-LPA engages in a salt-bridge interaction with the positively charged sidechain of K39^Nterm^, which remains stable for the first 250 ns (Figs 2B, 2C, and AC in S1 Appendix). In contrast, 18:0-LPA exhibits a transient breakage of this salt bridge around ~100–250 ns, with the interaction remained mostly intact for the remainder of the simulation (Figs 2D and AD in S1 Appendix). Both ligands maintain a stable hydrogen bond between the phosphate group and the sidechain -OH of Y34^Nterm^ throughout the 500 ns simulation. Within the extracellular loop (ECL1), the two ligands show notable differences: 18:1-LPA maintains a stable, high-occupancy interaction with T109^ECL1^ throughout the simulation (S1 Movie), while 18:0-LPA preferentially contacts T113^ECL1^, which persists for ~350 ns before the ligand reorients away from ECL1 (Figs 2C, 2E, AC and AD in S1 Appendix). The phosphate oxygen atoms of both ligands are additionally involved in salt bridge interactions with R124^3.28^ and K294^7.36^ from TM3 and TM7 (S2 Movie), respectively, with above-average occupancy rates (Fig 2C and 2E). This is consistent with mutagenesis studies showing that disruption of both residues impairs ligand binding [43]. Similar residue-ligand interaction patterns were observed in simulations without Gi protein (Fig D in S1 Appendix).

Beyond the polar headgroup interactions, the two ligands exhibit markedly distinct alkyl chain dynamics and hydrophobic contact profiles. Due to the high flexibility of both LPA species, they sampled multiple conformations away from their starting poses during the simulation. For 18:1-LPA, an initial internal angle (α, measured between the phosphate head P, the middle alkyl segment C9-C10, and the tail end C1-C2) of ~60° and a U-shaped alkyl chain evolved toward a more extended conformation with an internal angle of ~105° (Fig AE in S1 Appendix), providing a larger hydrophobic surface for interactions with pocket-lining residues. In contrast, 18:0-LPA adopted a nearly L-shaped conformation (internal angle, α ~ 75°), with the lower end of its alkyl chain pointing outwardly through the space between TM1 and TM7 toward the membrane bulk near ~400 ns (Fig 2D), reducing its interactions with ECL1 (Fig AD in S1 Appendix). These distinct conformational trajectories are reflected in the RMSD values: 18:0-LPA (7.7 ± 2.3 Å) was considerably more dynamic than 18:1-LPA (5.7 ± 1.1 Å), with the additional displacement toward TM7 partially accounting for this difference (Fig AF in S1 Appendix). At the level of residue contacts, the 18:0-LPA alkyl chain drifted toward TM1-TM2, acquiring contacts with V59^1.42^, C60^1.43^, and I63^1.46^ that are reduced or absent for 18:1-LPA (Fig B in S1 Appendix). In contrast, 18:1-LPA, with conformational restraints imposed by its cis double bond, showed greater contact with TM3 residues Q125^3.29^, D129^3.33^, and L132^3.36^, all of which have been implicated in agonist activity, as well as with W210^5.43^ at the pocket base. A particularly striking difference was observed at TM6: 18:1-LPA preferentially contacts W271^6.48^ of the C^6.47^W^6.48^xP^6.50^ motif, whereas 18:0-LPA interacts predominantly with P273^6.50^ of the same motif. Both ligands make extensive hydrophobic contacts with TM7 residues F296^7.38^, L297^7.39^, and A300^7.42^, with only subtle differences as outlined in the contact fingerprint (Figs B and C in S1 Appendix).

To quantify the energetic significance of these interactions, we used the MM/PBSA method to evaluate per-residue contributions to binding free energy. Residues forming polar interactions with the ligands, including K39^Nterm^, T109^ECL1^, T113^ECL1^, R124^3.28^, and K294^7.36^, contributed more substantially to relative binding energy for both ligands than hydrophobic residues (Fig 2G), consistent with the stable and persistent nature of these contacts described above. Hydrophobic residues (L105^2.60^, L132^3.36^, W210^5.43^, and W271^6.48^) exhibited more dynamic on-off-contact behavior, resulting in lower overall energetic contributions. R124^3.28^ and K294^7.36^ showed the highest contributions to binding affinity for both ligands, consistent with the mutagenesis data [27]. Among the differences, K39^Nterm^ contributed more to 18:0-LPA affinity than 18:1-LPA, correlating with the higher contact frequency of that residue in the 18:0-LPA simulations. Among hydrophobic residues, L297^7.39^ was the largest contributor for 18:0-LPA (-1.62 kcal/mol) while W210^5.43^ contributed more for 18:1-LPA (-1.67 kcal/mol). Overall, both ligands exhibited high relative binding affinity, with a slightly higher value for 18:0-LPA (-65.6 ± 4.2 kcal/mol) compared to 18:1-LPA (-63.9 ± 4.6 kcal/mol) (Table B in S1 Appendix). These results indicate that the greater agonist efficacy of 18:1-LPA is unlikely to be related to differences in binding affinity per se, but rather to the distinct conformational consequences of its alkyl chain geometry within the pocket.

### Insights on differential activation from dynamics of conserved motifs

**Fig 3:**
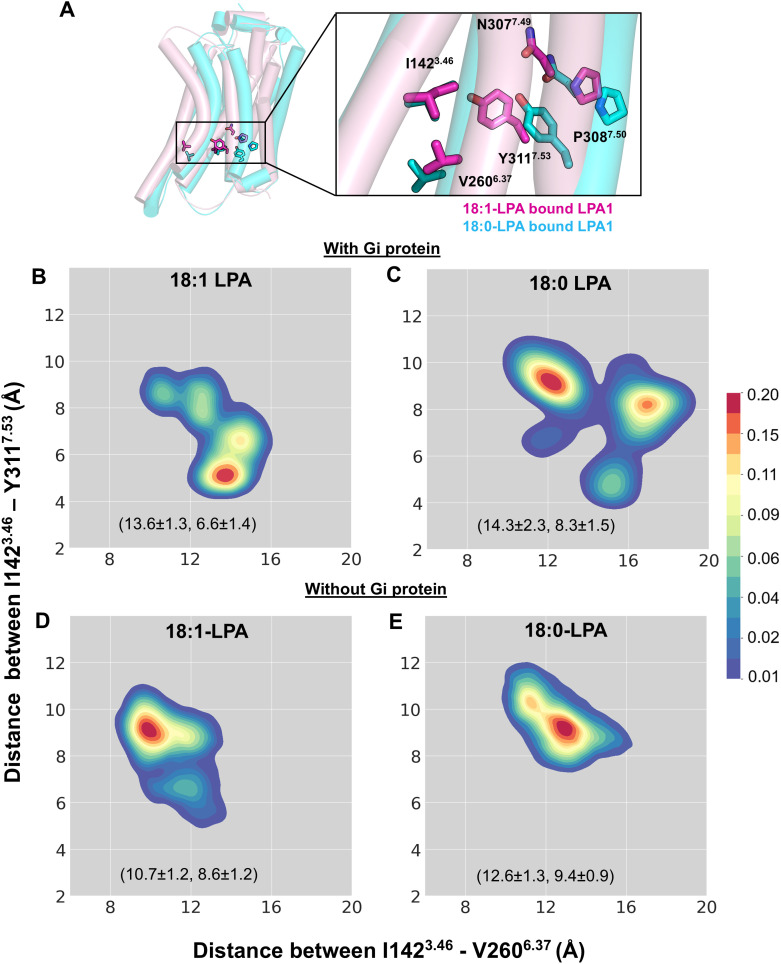
Differences in the interhelical activation-related distances between TM3-TM7 and TM3-TM6 of LPA1 provide the structural basis for full and partial agonist activity. **(A)** Critical residues of the activation switch, including the conserved NPxxY structural motif, TM3 (I142^3.46^), TM6 (V260^6.37^), and TM7 (Y311^7.53^), are shown from the superimposed cartoon representation of 18:0-LPA (cyan)- and 18:1-LPA (magenta)-bound LPA1 systems after 500 ns MD simulations. **(B-E)** 2D kernel density plots show the preferred TM3-TM6 and TM3-TM7 distances in four simulated systems. The closer interaction between TM3 (I142^3.46^) and TM7 (Y311^7.53^) is preferentially maintained in the 18:1-LPA-bound LPA1 with Gi (B) but not with the 18:0-LPA-LPA1-Gi system. The absence of Gi led to an increase in TM3-TM7 distance and a decrease in TM3-TM6 distance in both 18:1-LPA-LPA1 (D) and 18:0-LPA-LPA1 (E) receptor-only systems. All metrics in panels B-E aggregate all three 500-ns replicates per ligand.

Class A GPCRs have several conserved structural motifs and activation switches that exhibit marked differences upon activation due to ligand binding [30,44,45]. These motifs play critical roles in the transduction of ligand-initiated responses through a coordinated pattern of conformational changes and breakage or formation of specific interactions. Like other class A GPCRs, previous structural elucidation of both active and inactive structures of LPA1 has shown an outward movement of TM6 upon activation, while TM7 shifts inwardly towards TM3 (Fig 3A). As a result, Y311^7.53^ from the N^7.49^P^7.50^xxY^7.53^ conserved motif comes in close contact with I142^3.46^ at the intracellular ends of TM3, moving away from V260^6.37^ located on TM6. We assessed the movement and interactions of the three helices (TM3, TM6, and TM7) by quantifying the distances between the three residues during the entire simulation time in all replicates (Fig 3B-E).

Overall, in the 18:1-LPA-LPA1 system in complex with the heterotrimeric Gi protein, the most predominant conformational change of the receptor involves a closer distance of about 4–5 Å between Y311^7.53^ and I142^3.46^ and a greater distance of approximately 13 Å between I142^3.46^ and V260^6.37^ (Fig 3B). This implies that 18:1-LPA can restrict the conformation of the LPA1 receptor bound to the Gi protein in the active state for the cumulative simulation time of ~1.5 microseconds. Interestingly, in the absence of Gi, there was an outward movement of TM7, leading to an increase in the TM3-TM7 distance by ~4–5 Å and an inward movement of the TM6 by ~4 Å (Fig 3D). This difference is observed despite a similar binding pose and residue interactions of 18:1-LPA in the binding pocket of LPA1 with and without Gi protein.

With 18:0-LPA, LPA1 partially sampled a state with reduced TM3-TM7 distance (4–5 Å) and increased TM3-TM6 distance (15–16 Å) in the presence of heterotrimeric Gi protein. In the most dominant conformations, there was ~ 9 Å distance between TM3-TM7 (Fig 3C), which does not reflect a fully active state of LPA1. However, there was also an outward movement of the TM6 reflected by two clusters at ~12 Å and ~16 Å between TM3 and TM6; hence, the receptor is neither inactive. This observation likely reflects an intermediate state of the LPA1 receptor. Since 18:0-LPA is a partial agonist, it is unable to completely restrain the receptor in a fully active state despite the presence of the Gi protein (Fig 3C). Without the heterotrimeric Gi protein, 18:0-LPA maintains the receptor in intermediate states similar to those observed with Gi-protein (Fig 3E).

In addition to I142^3.46^-Y311^7.53^ distances, we analyzed ligand-induced differences in residue-residue distances at several other activation switches (Fig E in S1 Appendix). Importantly, in the presence of 18:1-

PA, V70^1.53^ shifts further away from Y311^7.53^ with an average distance of ~10Å which closely resembles the measured distance in the active LPA1 (PDB ID: 7TD1) structure (blue dotted line in Fig ED in S1 Appendix). Interestingly, this distance was maintained in both the presence and absence of the heterotrimeric Gi protein. In contrast, the presence of 18:0-LPA resulted in a closer distance between V70^1.53^ and Y311^7.53^ that more closely resembles the measured distance in the inactive (PDB ID: 4Z34) structure (red dotted line in Fig ED in S1 Appendix). These 18:0 LPA-induced effects were also independent of the presence of the Gi protein. Analyses of distances between E145^3.49^ and R146^3.50^ and L256^6.33^-R146^3.50^ revealed similar patterns among the two LPA species in the presence and absence of Gi protein (Fig EE-EF in S1 Appendix).

### Aromatic residue conformations support ligand-induced activation states

**Fig 4:**
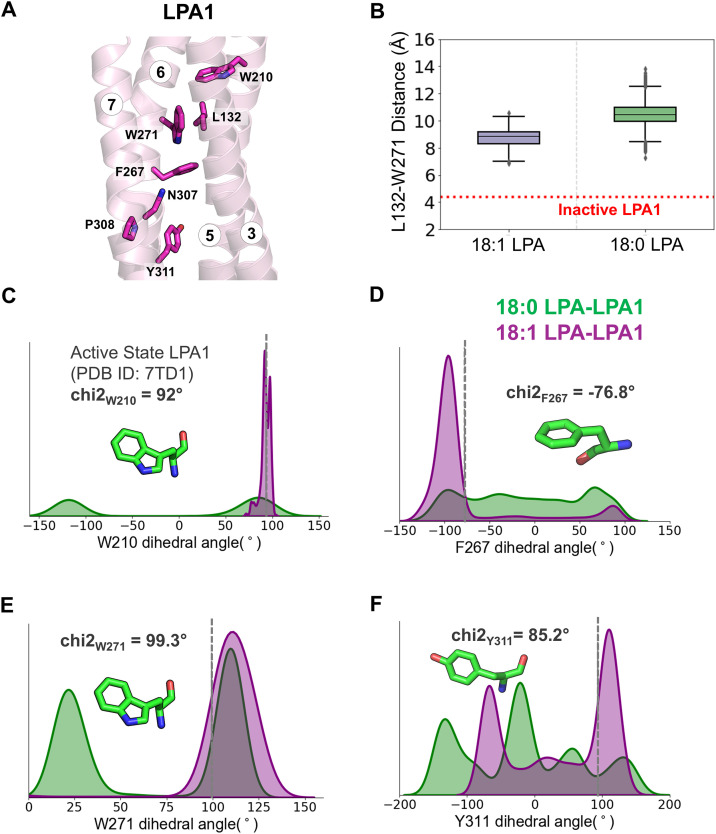
Ligand-induced conformational changes in residues that are part of conserved structural motifs at the base of the binding pocket. **(A)** Transmembrane helices 3, 5, 6, and 7 in a cartoon representation showing conserved residues forming specific structural motif interactions in LPA1. **(B)** The box plot shows the disrupted alkyl-π interaction distance between residue W271^6.48^ and L132^3.36^ in the presence of 18:0- (green) and 18:1-LPA (purple). This interaction appears to be intact with a distance of around 4Å in the inactive state (PDB ID 4Z35), as indicated by the dotted red line. **(C-F)** Density plots show the chi2 dihedral angles for W210^5.43^, F267^6.44^, W271^6.48^ and Y311^7.53^ for both 18:0-LPA- (green) and 18:1-LPA-bound LPA1 (purple). In each plot, the dotted vertical line shows the corresponding value (also given as text) observed in the active state LPA1 (PDB ID 7TD1). Distributions reflect all three replicates per system.

The C^6.47^W^6.48^xP^6.50^ motif is located near the floor of the orthosteric binding site in LPA1 [46] (Fig 4A). In the inactive structure (PDB ID: 4Z34), L132^3.36^ forms a notable alkyl-π interaction with W271^6.48^ from the CWxP motif at a distance of ~4.3 Å, indicated by a dotted line in Fig 4B. This interaction is absent in the active structure (PDB ID: 7TD1) in the presence of an agonist. This disruption appears to be partly due to the agonist-induced rotameric switching of neighboring W210^5.43^ at the base of the pocket. The chi2 dihedral angle of W210^5.43^ is 92 and -100 degrees in the active and inactive states, respectively (Table C in S1 Appendix). In our simulations, the average chi2 dihedral angles of W210^5.43^ in the presence of 18:0-LPA and 18:1-LPA are 80.6 ± 11.8 and 92.9 ± 6.6 degrees, respectively, which are near the active state conformation of LPA1 (Fig 4C).

18:0-LPA and 18:1-LPA differ significantly in their ability to maintain other key activation switches, such as W271^6.48^, F267^6.44^, and Y311^7.53^ of conserved structural motifs, C^6.47^W^6.48^xP^6.50^, P^5.50^I^3.40^F^6.44^, and N^7.49^P^7.50^xxY^7.53^, in active state conformations. In the presence of 18:1-LPA, the chi2 dihedral angles of F267^6.44^ and W271^6.48^ are around -95^o^ and 106^o^, respectively, and remain stabilized around the active state conformations (Fig 4D and 4E and Table C in S1 Appendix) [27]. However, in the presence of 18:0-LPA, the mean dihedral angle of W271^6.48^ is around 27 ^o^ and there is a limited sampling around the active state conformation (Fig 4E). Also, the dihedral angle of F267^6.44^ shows wide variations (-3.9 ± 71.9) with two clusters, likely representing intermediate states (Fig 4D). In one of the clusters, the conformation of F267^6.44^ is synonymous with the active state, while in the other cluster, the F267^6.44^ dihedral is similar to the inactive state (Table C in S1 Appendix).

Interestingly, within the N^7.49^P^7.50^xxY^7.53^ motif, Y311^7.53^ underwent significant conformational changes in the presence of 18:0- and 18:1-LPA, resulting in a bimodal distribution of its dihedral angles in both systems. However, the distribution toward the active state is more prominent in the presence of 18:1-LPA than in the presence of 18:0-LPA (Fig 4F). In the case of P308^7.50^, both 18:0- and 18:1-LPA allowed the receptor to sample the dihedral angles mostly near the active state, although the angles appeared in the inactive state to some extent (Fig F in S1 Appendix). Together, the differential modulation of these conserved motifs in the presence of 18:0- and 18:1-LPA provides structural basis for their distinct effects on the receptor-Gi protein interface and downstream ERK activation.

### Ligand-induced conformational changes affect LPA1 and Gi protein interactions

**Fig 5:**
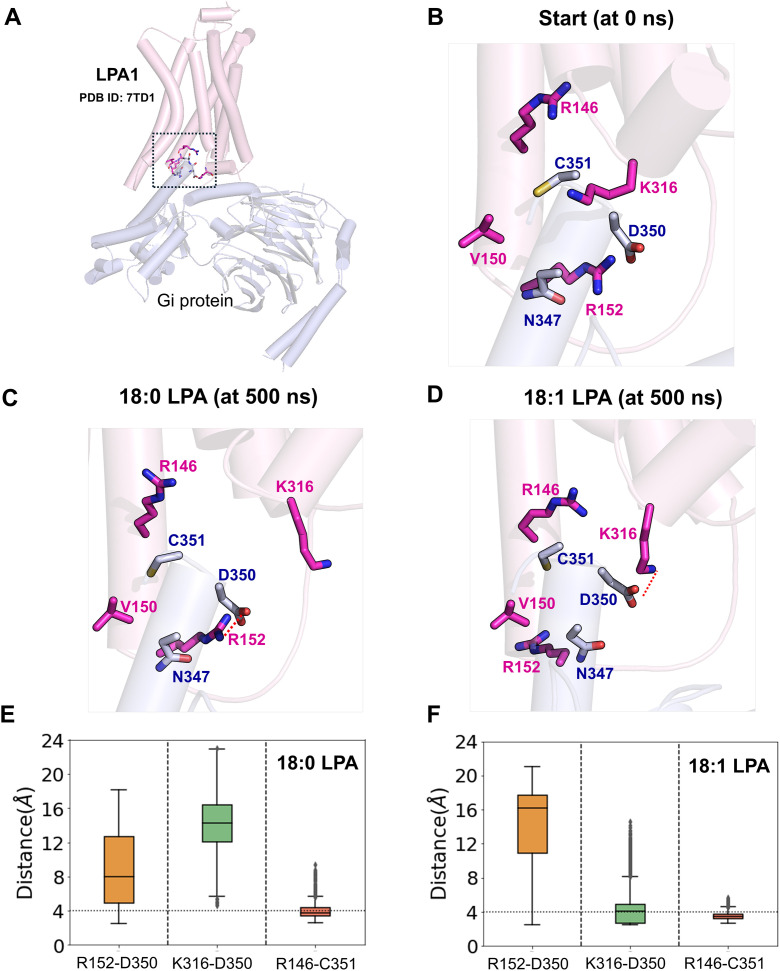
18:0- and 18:1-LPA distinctly affect LPA1-Gi protein interactions. (A) Dynamics and critical residue interactions at the LPA1-Gi protein interface in the presence of two LPA species at the beginning (time = 0 ns) and end of 500 ns MD simulations. LPA1 and Gi protein are shown in cartoon representations in pink and light blue; critical residues of the respective interacting species are shown in licorice in magenta and blue, respectively. (B and C) Boxplots of distances between critical residues forming important polar interactions at the interface between LPA1 and Gα-helix of the G-protein in 18:0-LPA and 18:1-LPA bound systems, respectively. The strong salt bridge between K316^H8^ and D350^G.H5.22^ observed in the presence of 18:1-LPA is absent in the 18:0-LPA system.

In GPCRs, ligand-induced receptor activation signals are transmitted through a series of activation switches, eventually resulting in changes at the intracellular interface that facilitate and maintain G-protein coupling and interactions (Figs 5A and GA in S1 Appendix). Our MD simulation results are in agreement with the recent cryo-EM study (PDB ID: 7TD1) that the receptor interface in LPA1 includes the intracellular ends of TM3-TM6 as well as the various intracellular loops, especially ICL2 [27,28]. These regions are involved in very extensive polar interactions, particularly salt bridges with the C-terminal end of the G_αi_-α5 helix of the G-protein (S3 and S4 Movies).

One of the important determinants of the receptor-Gi protein-coupling is the hydrogen bond between R146^3.50^ (from the E^3.49^R^3.50^H^3.51^ motif) and the backbone carbonyl atom of C351^G.H5.23^ from the Gα-helix. In both the 18:0- and 18:1-LPA-LPA1 systems (Fig 5B and 5C), this interaction was maintained entirely during the 500 ns simulations (Fig 5C and 5D). In addition, C351^G.H5.23^ engages in polar interactions with T149^3.53^ and V150^3.54^ residues at the intracellular ends of TM3. These interaction profiles were similar with both ligands (Fig GB and GC in S1 Appendix).

Not surprisingly, the differential movement of the TM6 and TM7 helices, as outlined earlier, results in distinct alterations at the receptor-Gi-protein interface. For example, the cryo-EM structure of LPA1-LPA (PDB ID:7TD1) features a salt bridge between R152^3.56^ from the receptor and D350^G.H5.22^ of the G-protein. This interaction was disrupted to a much greater extent in the 18:1 simulation than in the 18:0-LPA -LPA1 system (Fig 5C and 5D). The differential movement of R152^3.56^ resulted in differential contact with I343^G.H5.15^ of the Gα helix in the 18:0-LPA bound systems (Fig GB and GC in S1 Appendix). However, the strong polar interaction of R152^3.56^ with N347^G.H5.19^ was well preserved in both systems.

In the 18:1-LPA-LPA1 simulations, the sustained inward movement of TM7 maintained a very stable interaction between K316^H8^ at the intracellular end of the receptor and D350^G.H5.22^ in the Gα-helix (Fig 5B and 5C). This interaction was noticeably absent in 18:0-LPA-LPA1 simulations. D350^G.H5.22^ (Gα-helix) contacts R146^3.50^ to a greater extent in the 18:1-LPA-LPA1 simulations than in the 18:0-LPA simulations (Fig GB and GC in S1 Appendix).

The intracellular loops of GPCRs have been shown to participate to varying extents in G-protein coupling. Differential interaction with the ICL3, which occurs with the S1P1 receptor, was previously proposed as a difference with the LPA1 interface, where these interactions are less significant. In our simulations with 18:0- and 18:1-LPA, the ICL2 residues, including M153^ICL2^ and Q154^ICL2^, are involved in hydrophobic interactions with I343^G.H5.15^ for the majority of the simulation time. In 18:1-LPA simulations, Q154^ICL2^ also engages in significant polar interactions with N347^G.H5.19^ that were less prevalent in 18:0-LPA simulations (Fig GB and GC in S1 Appendix). In addition, hydrophobic interactions between L256^6.33^ in TM6 and L353^G.H5.25^ of the Gα-helix were observed as common to both systems. TM5 residues, including R235^5.68^, R238^5.71^, and M239^5.72^, also play a role in G-protein coupling via polar and hydrophobic interactions with F334^G.H5.06^, D337^G.H5.09^, and D341^G.H5.13^ of Gi protein (Fig GB and and GC in S1 Appendix).

### Formation of water channels across the transmembrane helices

**Fig 6:**
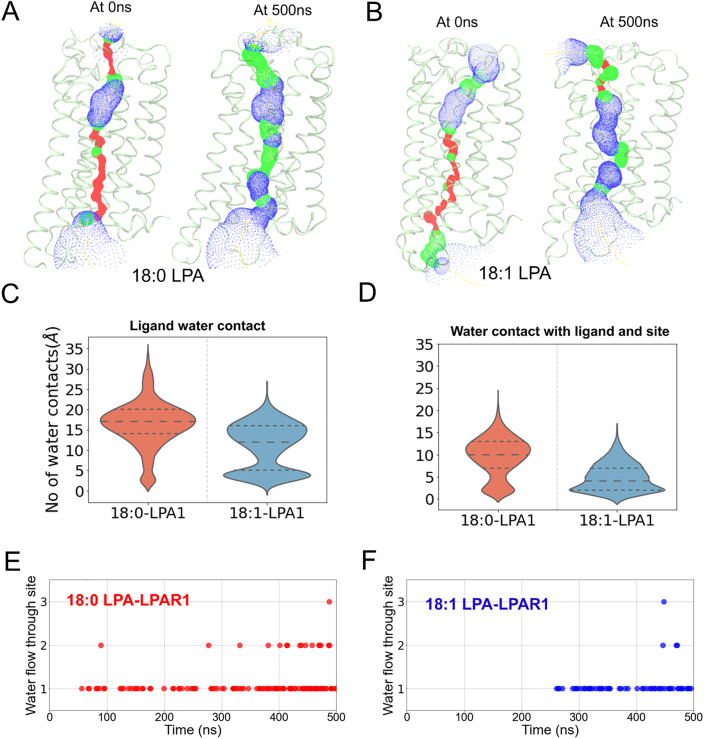
18:0- and 18:1-LPA induce the formation of water channels in LPA1. (A-B) Opening of an intracellular water channel along the transmembrane helices during the simulation in 18:0-LPA (A) and 18:1-LPA bound LPA1-Gi-protein systems. (C) Ligand-water contacts within the receptor. This was measured by computing the total number of water molecules within 4 Å of 18:0- (red) and 18:1-LPA (blue) species. (D) Water molecules that are in contact with both the binding pocket residues and the ligand. (E-F) The number of water molecules that flow from the extracellular part of the receptor through the base of the binding pocket in 18:0-LPA (E) and 18:1-LPA (F) bound LPA1.

Recent studies have shown that water networks could modulate signal transduction from the extracellular region to the intracellular loop regions of GPCRs. Specifically, the presence of large water clusters near the intracellular region of TM7 N^7.49^P^7.50^xxY^7.53^ motif has been observed in mu-opioid, β-adrenergic, and adenosine receptors [47–49]. In our LPA1 simulations with both 18:0- and 18:1-LPA, we observed the gradual expansion of continuous water channels that run from the extracellular to intracellular ends of the receptor (Fig 6A and 6B). Particularly, there was a bulge in the channel at the intracellular end corresponding to the region of the N^7.49^P^7.50^xxY^7.53^ motif. This differs from the inactive LPA1 structure that has been reported to have a discontinuous channel, likely due to water occlusion in the TMD areas [50]. To confirm the likelihood that this channel conducts water molecules, we measured water flux between the upper and lower ends of the binding pocket (Fig 6E and 6F). In both systems, at least one water molecule moves between the upper and lower regions of the receptor for a significant period during the simulation time. Water flow through the channel was slightly greater in the 18:0-LPA system compared to 18:1-LPA; this is consistent with the observation that the 18:0-LPA-LPA1 system features a more extensive water channel (Fig 6A and 6B) between the intracellular and extracellular regions.

Beyond the transmembrane water channel, ligand-water contacts were also monitored throughout the simulation. Overall, water contact with the ligands was higher for 18:0-LPA than 18:1-LPA (Fig 6C) consistent with its more extensive channel described above. Initially, water contacts were predominantly with the glycerol-phosphate groups, with little to no water contact with the alkyl chains (Fig HA and HB in S1 Appendix). Alkyl chain water contact emerged progressively as the water network expanded through the binding site. While mobile water molecules were observed in the orthosteric site in the presence of both ligands (Fig 6D), no specific lasting water-mediated ligand interactions were identified.

### Optimal communication paths between the activation site and G-protein coupling interface

To obtain insights into the signal transduction pathway between orthosteric site residues and residues that interface with Gi-protein at the intracellular side, we carried out dynamic network analyses using the VMD plugin Network View. The optimal residue-to-residue communication paths between crucial binding site residues (R124^3.28^, W271^6.48^, and K294^7.36^) and residues that engage with the Gi-protein interface (R146^3.50^, R152^3.56,^ and K316^H8^) were analyzed based on the MD trajectory data.

**Fig 7:**
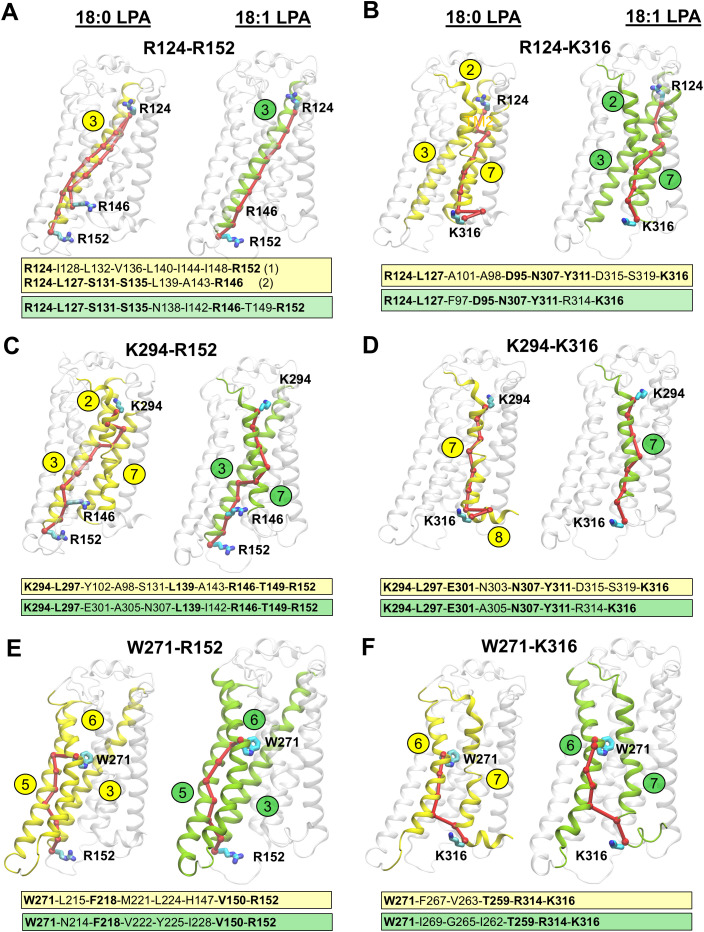
Optimal signal transduction paths in 18:0-LPA- and 18:1-LPA- bound LPA1 systems, as determined by network analyses. The optimal paths were calculated between critical binding site residues and residues at the Gi-protein coupling interface. In each case, 18:0-LPA- and 18:1-LPA-activated LPA1 receptors are depicted in cartoon representations and participating helices are colored and labeled in yellow and green, respectively. A-B) The optimal paths between the orthosteric site residue R124^3.28^ and residues at the Gi protein-coupling interface R152^3.56^ and K316^H8^, respectively. C-D) The optimal paths between K294^7.36^ with R152^3.56^ and K316^H8^, respectively. E-F) The optimal paths between W271^6.48^ and R152^3.56^ and K316^H8^, respectively. For each path, all the residues along the entire path for 18:0- and 18:1-LPA bound LPA1 are given in yellow and green text boxes, respectively. Common residues among the paths are in bold fonts.

In the 18:1-LPA bound LPA1-Gi protein system, a single optimal path connected R124^3.28^ in TM3 with R146^3.50^ and R152^3.56^, which directly interact with the Gα helix of the Gi protein (Fig 7A). This path spans only the TM3 helix and includes I142^3.46^, which was noted earlier as having closer contact with Y311^7.53^ in the 18:1-LPA-LPA1 system compared to the 18:0-LPA-LPA1 system (Fig 4A and 4F). However, in the 18:0-LPA-LPA1 system, two optimal paths connect R124^3.28^ simultaneously to R146^3.50^ and R152^3.56^. The path running from R124^3.28^-R146^3.50^, including residues (L127^3.31^, S131^3.35^, and S135^3.39^), resembles that of the 18:1-LPA-LPA1 receptor system more closely than the R124^3.28^-R152^3.56^ path. The mechanistic implication is that ligand contacts at R124^3.28^ propagate conformational strain along TM3 through I142^3.46^, positioning R146^3.50^ and R152^3.56^ at the intracellular face for productive Gα engagement. In the 18:1-LPA system, the compact single-path geometry reflects a more rigid TM3 conformation that directly supports the active-state R146^3.50^-C351^G.H5.23^ hydrogen bond and the R152^3.56^-D350^G.H5.22^ salt bridge discussed above. In the 18:0-LPA system, the bifurcated path with additional intermediate residues (L127^3.31^, S131^3.35^, S135^3.39^) suggests a less efficiently coupled TM3, consistent with the partial disruption of the R152^3.56^-D350^G.H5.22^ interaction observed in the Gi-protein interface analyses.

The optimal communication path between R124^3.28^ and K316^H8^ passes along TM2, TM3, and TM7 helices in both 18:0- and 18:1-LPA bound systems. One common residue along this path, D95^2.50^, is conserved across all LPA (1–6) receptors (Figs 7B and I in S1 Appendix). Interestingly, this path also included N307^7.49^ and Y311^7.53^ from the N^7.49^P^7.50^xxY^7.53^ motif in both systems with 18:0 and 18:1-LPAs. The noticeable difference here is the presence of R314^7.56^ in the 18:1-LPA1 system, unlike the 18:0-LPA1 system, which includes D315^H8^ and S319^H8^ from the helix 8.

Also, the residue communication path between K294^7.36^ and R152^3.56^ transverses TM3 and TM7 in the 18:1-LPA1 system. Similar to the R124^3.28^-R152^3.56^ path, it included residue I142 from TM3. The path also features N307^7.49^ from the N^7.49^P^7.50^xxY^7.53^ motif. In contrast, this same path (K294^7.36^-R152^3.56^) in the 18:0-LPA liganded system also transverses TM2 (A98^2.53^ and Y102^2.57^) and did not pass through the I142^3.46^ residue. Across all K294^7.36^ paths with the G-protein interface residues (R152^3.56^ and K316^H8^), L297^7.39^, located near the bottom of the binding site, is the most commonly occurring residue (Fig 7C and 7D).

Unlike the earlier connections analyzed, the optimal residue communication path between W271^6.48^-R152^3.56^ passes through the TM5 in the presence of both ligands (Fig 7E and 7F). The common residue here, F218^5.51^, is conserved across the EDG LPA (1–3) receptors. This path also included V150^3.54^, which interacts with the Gi protein in both 18:0-LPA1 and 18:1-LPA receptor systems. Also, the W271^6.48^-K316^H8^ path noticeably did not pass through the N^7.49^P^7.50^xxY^7.53^ motif with either 18:0- or 18:1-LPA ligands bound to the LPA1 receptor. The only common residues with both ligands across this path include T259^6.36^ and R314^7.56^. The uniqueness of the W271^6.48^ to G-protein interface communication path prompted us to evaluate its optimal connection pathway with site residues (R124^3.28^, K294^7.36^) and the N^7.49^P^7.50^xxY^7.53^ motif. Consequently, we observed significantly different connections of W271 to (R124^3.28^, K294^7.36^, and Y311^7.53^) between 18:0-LPA and 18:1-LPA bound LPA1 receptor systems (Fig IA-IC in S1 Appendix).

### Access and binding mechanisms of 18:0 and 18:1-LPAs to LPA1

**Fig 8:**
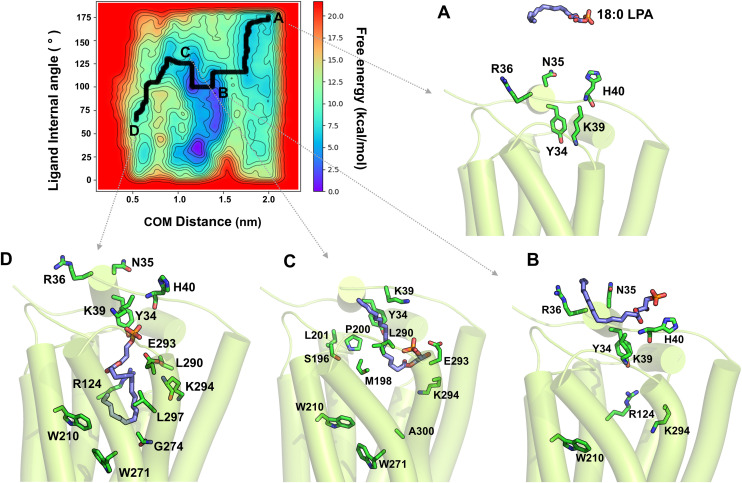
The free-energy surface (FES) of 18:0-LPA’s access and binding to the LPA1 receptor from the aqueous phase. The 2D FES is depicted using two collective variables: 1) the center-of-mass distance between the ligand and binding site residues (x-axis) and 2) the ligand’s internal angle α (y-axis). The minimum energy path of the ligand to the receptor, as determined by WT-metaD, is depicted in the bold black line. **(A-D)** Representative starting, intermediate, and bound conformations are shown. (A) 18:0-LPA is initially located ~20 Å away from the receptor in the aqueous phase. (B) 18:0-LPA rapidly approaches the receptor’s N-terminal loop; the phosphate head group of the ligand contacts K39 of the N-terminal first and forms polar interactions with K39^Nterm^, H40^Nterm^, Y34^Nterm^, and R36^Nterm^. **(C)** The 18:0-LPA alkyl chain undergoes significant conformational changes and attempts to enter the receptor via the space between the ECL1 and ECL2 loops. **(D)** The pouch-like shape of the binding pocket forces the 18:0-LPA alkyl chain to bend, forming hydrophobic contacts with R124^3.28^, Q125^3.29^, G274^6.51^, E293^7.35^, and K294^7.36^ in TM3 while maintaining polar contacts with N-terminal Y34^Nterm^.

**Fig 9:**
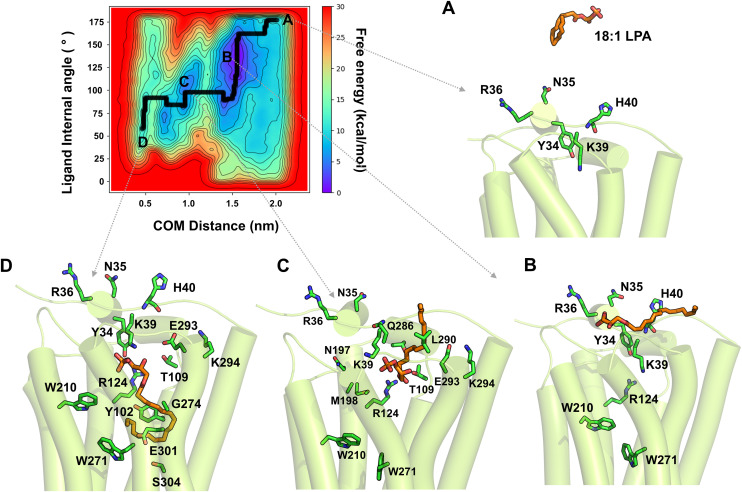
The free-energy surface (FES) of 18:1-LPA’s access and binding to LPA1. The 2D FES is depicted using two collective variables: the distance between the center-of-mass of the ligand and that of binding site residues (x-axis) and the ligand’s internal angle α. The minimum energy path of the ligand to the receptor is represented by the bold black line, and major intermediate conformations are labeled A-D. (A) 18:1-LPA is initially located within the aqueous bulk at a distance of ~20 Å from the receptor. (B) 18:1-LPA rapidly approaches the N-terminal loop of the receptor. While the phosphate head group first contacts K39^Nterm^ in the N-terminal and forms polar interactions with K39^Nterm^, H40^Nterm^, and R36^Nterm^, the alkyl chain partitions into the membrane. (C) 18:1-LPA enters the receptor via the extracellular loops. **(D)** As 18:1-LPA approaches the binding site, its lower alkyl chain comes in contact first with L290^7.32^ and E293^7.35^. The ligand adopts a final pose with a bent alkyl conformation.

To investigate the ligand access routes and binding to the orthosteric binding pocket, we performed WT-metaD association simulations in triplicate for each ligand, starting from the extracellular aqueous phase at ~20 Å from the binding site residues. The resulting free-energy surfaces (FES), characterized using the ligand-site center-of-mass (COM) distance and ligand internal angle (α) as collective variables, are shown in (Figs 8, 9, and J in S1 Appendix) for 18:0-LPA and 18:1-LPA, respectively. Full simulation details are provided in the Methods section.

Both ligands approached the receptor from the extracellular aqueous phase and rapidly engaged the N-terminal loop prior to entering the orthosteric pocket (Figs 8A and 9A) [51]. The polar glycerol-phosphate headgroup anchored to N-terminal residues Y34^Nterm^, N35^Nterm^, R36^Nterm^, and K39^Nterm^ in both cases, with the alkyl chain curling to minimize its solvent-exposed surface area while these interactions were established (S5 and S6 Movies). Notably, 18:0-LPA maintained these N-terminal contacts for nearly 40% of the total simulation time (~15 ns) before entering the pocket (Fig 8B), whereas 18:1-LPA transitioned more rapidly toward the pocket entrance, additionally making sparse contact with H40^Nterm^ (Fig 9B). In both ligands, the N-terminal polar interactions were sustained throughout the entry process, consistent with the established role of Y34^Nterm^ and K39^Nterm^ as key gatekeepers of ligand access to LPA1 [50].

As 18:0-LPA approached the pocket entrance, it underwent a rapid conformational change from an extended orientation approximately parallel to the membrane plane to a nearly perpendicular acute orientation, reflected in a sharp decrease of its internal angle α from ~160° to ~60° (Fig 8C). The alkyl chain first contacted ECL2 residues P200^ECL2^ and M198^ECL2^, subsequently engaging S196^ECL2^ and L201^ECL2^, with the ligand entering predominantly through the space between the ECL loops closer to ECL2. Within the transmembrane region, initial contacts were established with TM7 residues L290^7.32^ and E293^7.35^, followed by deeper penetration into the pocket with the alkyl chain contacting R124^3.28^, G274^6.51^, K294^7.36^, and L297^7.39^ from TM3, TM6, and TM7 (Fig 8C and 8D). In its final bound orientation, 18:0-LPA adopted a bent alkyl conformation pushing against TM7, while the phosphate headgroup maintained polar contacts with E293^7.35^ and a hydrogen bond with Y34^Nterm^ (Fig 8D).

18:1-LPA also entered through the spaces between the extracellular loops, though with a broader set of initial ECL contacts than 18:0-LPA, including P200^ECL2^, M198^ECL2^, N197^ECL2^, T109^ECL1^, and Q286^ECL3^ (Fig 9C). Similarly, the alkyl chain underwent a significant conformational change upon entry, with the internal angle α decreasing sharply from ~150° to ~60° (Fig 9B and 9C). Within the transmembrane region, the ligand established contacts with L290^7.32^, E293^7.35^, and K294^7.36^ along the entry path. However, the final bound pose of 18:1-LPA is distinctly different from that of 18:0-LPA: the alkyl chain established hydrophobic contacts with Y102^2.57^, R124^3.28^, W210^5.43^, W271^6.48^, G274^6.51^, E301^7.43^, and S304^7.46^ (Fig 9D), engaging aromatic residues at the pocket base that are critical for activation switch modulation. Additionally, the ligand formed a new hydrogen bond with T109^ECL1^ in its final pose, while sustaining polar interactions with Y34^Nterm^ and K39^Nterm^.

### Membrane Partitioning Characteristics of 18:0 and 18:1-LPA Species

Previous studies from our lab indicated that despite having similar lipophilic-amphiphilic characteristics, ligands can exhibit distinct membrane partitioning characteristics and take separate paths to reach the orthosteric sites in class A GPCRs [52]. Therefore, to elucidate the likely differences in membrane partitioning characteristics between 18:0-LPA and 18:1-LPA (Fig KA in S1 Appendix), we used steered molecular dynamics (SMD) and umbrella sampling (US) simulations of the ligands in a model membrane consisting of 1-palmitoyl-2-oleyl-sn-glycero-3-phosphocholine (POPC) and cholesterol. The potential of mean force (PMF) curve (Fig KB in S1 Appendix) derived from the US simulations revealed the free energy of solvation for both ligands partitioning from water into the membrane along the bilayer normal (z-axis). The free-energy minima and maxima on the PMF curve indicate the most and least energetically favorable locations for the ligands. Although both ligands show favorable partitioning energy from water to the membrane, the phase transfer process for 18:0-LPA (ΔG_partitioning_ = -9.3 ± 0.05 kcal/mol) appeared to be more energetically favorable than 18:1-LPA (ΔG_partitioning_ = -7.2 ± 0.07 kcal/mol). Interestingly, the free-energy minima of the center-of-mass of both ligands (Table D in S1 Appendix) are located at |Z _min_| ~ 15 Å. Both ligands encountered an energetic barrier as they approached the membrane core. However, the energy barrier is relatively higher for 18:1-LPA (ΔG_crossing_ = 12.4 ± 0.08 kcal/mol) as compared to 18:0-LPA (ΔG_crossing_ = 11.1 ± 0.06 kcal/mol). For both ligands, the thermally accessible regions (RT = 0.616 kcal/mol, temperature = 310 K) extended for ≈ 2Å on either side of Z_min_.

To obtain further insights into the differences observed in the solvation-free-energy profile of both ligands, we analyzed the ligand internal angle α within the bilayer. Not surprisingly, both ligands adopt a nearly extended conformation. The average conformation of 18:0-LPA (145.4^o^ ± 11.2^o^) is such that the head tilts acutely towards the membrane bilayer normal while the alkyl chain adopts a nearly straight orientation in the membrane core to maximize its hydrophobic interactions with the lipid tails (Fig KC and KD in S1 Appendix). The internal angle of 18:1-LPA is slightly lower (139.1^o^ ± 12.5^o^) than 18:0-LPA. The probability distribution plot (Fig KD in S1 Appendix) also showed that 18:1-LPA explored more conformations in the membrane than 18:0-LPA. This is evident in the relatively higher standard deviation.

Also, we calculated the atomic contacts (as % contact frequency), which indicates the fraction of the simulation time during which different parts of 18:0-LPA and 18:1-LPA are within 4 Å of the various functional groups of the lipids. These analyses were done using the simulation trajectory representing the low-energy windows |Z_min_| ~ 15 ± 2 Å for both ligands (Fig L in S1 Appendix). In both ligands, the phosphate headgroup maintained strong contact with the choline functional groups of the lipids while staying further away from the glyceryl carbonyl and alkyl lipid groups. However, there was a slightly higher frequency in 18:1-LPA’s phosphate group contact with the choline lipid group. The glycerol backbone in both ligands had a high frequency of contact with all the lipid headgroups (choline, phosphate, glyceryl carbonyl). Again, the glycerol backbone of 18:1-LPA had a slightly greater contact than 18:0-LPA. Interestingly, a similar observation was noted in the ligand’s alkyl chain interaction, where both the upper and lower parts of the alkyl chain of 18:1-LPA had a higher frequency of contact with the lipid’s alkyl group than the alkyl chain of 18:0-LPA. Overall, 18:0 LPA and 18:1 LPA have very similar membrane partitioning profiles. Although the free energy of partitioning seems slightly more favorable for 18:0-LPA, 18:1-LPA explored more conformations and contacts with the membrane functional groups.

## Discussion

The present study provides a mechanistic explanation for how a single unsaturation in 18:1‑LPA, relative to the saturated 18:0‑LPA, reshapes the activation landscape of LPA1 and leads to markedly different cellular outcomes. Rather than affecting overall affinity, which is similar or slightly higher for 18:0‑LPA, the double bond shifts how the ligand engages the lower portion of the orthosteric pocket, producing distinct effects on conserved activation switches and on the intracellular architecture required for productive Gi coupling. The convergence of biochemical data with ensemble-level simulations allows us to define a functional model in which efficacy arises primarily from the stabilization of activation‑competent receptor ensembles rather than from binding strength.

Across replicates and simulation conditions, both ligands maintain comparable head‑group interactions, but the shape imposed by the C9 = C10 double bond alters how the alkyl chain samples the base of the pocket. This geometric distinction positions 18:1-LPA to engage aromatic and hydrophobic features that facilitate productive rearrangements in well‑established GPCR microswitches. In contrast, the greater flexibility of the saturated tail in 18:0‑LPA encourages configurations that partially oppose these movements. Thus, the efficacy difference is not due to which residues are contacted, but how the ligand’s shape biases the receptor’s conformational ensemble.

In GPCRs, receptor activation and G‑protein coupling reflect the population of conformational states rather than the attainment of a single structural endpoint. The present results align with this view: 18:1‑LPA enriches ensembles with intracellular geometries that resemble active Gi‑coupling states, whereas 18:0‑LPA favors intermediate states that do not fully support the same coupling effectiveness. Importantly, these differences emerge without large differences in binding energy and without altering the fundamental head‑group recognition shared by both ligands. The data therefore support a model in which subtle ligand-specific biases at the pocket base propagate through conserved activation motifs to tune the intracellular interface.

These findings are grounded in and extend the structural insights provided by recent cryo-EM studies of LPA1. The active-state structures of LPA1 bound to 18:1-LPA (PDB: 7TD1) and a potent non-lipid agonist revealed the orthosteric pocket architecture and identified key polar contacts at Y34^Nterm^, R124^3.28^, and K294^7.36^ as essential for ligand recognition and receptor activation^27, 28^. Our simulations confirm and dynamically elaborate these observations: the salt bridges with R124^3.28^ and K294^7.36^ are the highest-energy contributors to binding affinity for both LPA species, consistent with mutagenesis data showing that their disruption abolishes Gi coupling [43]. The inactive-state structures (PDB: 4Z34, 4Z35) established that activation involves an outward movement of TM6 and inward movement of TM7, accompanied by rotameric transitions in the CWxP and PIF motifs [26]. Our simulations show that 18:1-LPA, but not 18:0-LPA, robustly stabilizes these transitions, specifically the W271^6.48^ rotamer shift and the disruption of the L132^3.36^-W271^6.48^ alkyl-π interaction, linking pocket-level chemistry directly to the activation switch rearrangements inferred from static structures. The observation that efficacy is encoded at the level of alkyl chain geometry rather than head-group chemistry is consistent with the chain-length activity relationships reported by Troupiotis-Tsaïlaki et al., who showed that lipid chain geometry drives activation of lipid GPCRs [25]. More broadly, our results align with the conformational selection model of GPCR activation proposed by Zhou et al., in which agonist efficacy reflects the degree to which a ligand stabilizes the receptor in activation-competent states rather than the magnitude of binding affinity [30,53].

Several limitations of this study should be acknowledged. First, no experimental structure of LPA1 bound to 18:0-LPA is currently available, requiring us to rely on molecular docking to generate the starting pose for 18:0-LPA simulations. While the docked pose is consistent with known binding site interactions and yields stable MD trajectories, the absence of an experimental reference means that subtle differences in the initial binding geometry cannot be fully excluded as a source of bias. Second, both LPA species carry a highly charged phosphate head group that engages predominantly positively charged residues (R124^3.28^, K294^7.36^, K39^Nterm^). The CHARMM36 and CGenFF force fields used here assign static partial charges and do not account for electronic polarization effects that may modulate these strong electrostatic interactions. Polarizable force fields such as CHARMM Drude would in principle provide a more physically rigorous treatment of these contacts. However, given the consistency of our results across independent replicates and the agreement of key interaction patterns with mutagenesis and cryo-EM data, we expect that the central conclusions regarding differential alkyl chain dynamics and activation switch modulation would be robust to this approximation.

## Conclusions

This study provides a mechanistic basis for the differential efficacy of 18:0-LPA and 18:1-LPA at the LPA1 receptor, demonstrating that a single C9 = C10 double bond, despite negligible impact on binding affinity, fundamentally reshapes how the ligand biases the receptor’s conformational ensemble. The geometric constraint imposed by the cis double bond positions 18:1-LPA to engage W271^6.48^ and F267^6.44^ of the conserved CWxP and PIF activation motifs, stabilizing TM3-TM7 active-state geometry and promoting a productive LPA1-Gi protein interface, most critically the K316^H8^-D350^G.H5.22^ interaction. In contrast, the greater conformational freedom of the saturated 18:0-LPA alkyl chain leads to preferential contact with P273^6.50^, insufficient stabilization of these switches, and an intermediate receptor conformation that only partially supports Gi coupling, consistent with its partial agonism profile. Both ligands share similar membrane partitioning characteristics and access the orthosteric site through aqueous routes, with N-terminal residues Y34 and K39 serving as key gatekeepers. Together, these findings establish that ligand efficacy at lipid GPCRs can be encoded by subtle geometric differences in the hydrophobic portion of the ligand, with implications for the rational design of LPA1-selective modulators.

## Materials and methods

### In vitro studies

*Materials:* LPA species were obtained from the following sources: (18:1; oleoyl) from Echelon Biosciences (Salt Lake City, UT), and LPA (18:0; stearoyl) from Avanti Polar Lipids (Birmingham, AL). LPAs were prepared in a stock solution with 4 mg/ml fatty-acid bovine serum albumin (BSA), stored at -20^o^C, and delivered to cells as a 1000X stock solution. Vehicle controls for LPA had a final concentration of 4 µg/ml BSA. Anti-phospho-Erk (#9106) was purchased from Cell Signaling Technologies (Danvers, MA) and used at 1:1000 dilution. Anti-GAPDH (sc-25778), obtained from Santa Cruz Biotechnologies (Dallas, TX), was used at 1:2000 dilution. Anti-rabbit IgG secondary antibody (#Lot 31) and Anti-mouse IgG, HRP-linked Antibody (#7076) were purchased from Cell Signaling Technologies (Danvers, MA) and used at 1:2000 dilution.

*Cell culture:* PC-3 cells were obtained from the American Type Culture Collection (Manassas, VA). The cells were grown in RPMI 1640 medium (Cytiva, Marlborough, MA) with 10% FBS (VWR, Visalia, CA), on standard tissue culture plastic in an incubator maintained at 37˚C with 5% CO2.

*Immunoblotting:* Cells were rinsed twice with ice-cold phosphate-buffered saline (PBS), harvested by scraping into 1 ml ice-cold PBS, collected by centrifugation at 10,000xg for 10 min at 4°C, and resuspended in ice-cold lysis buffer (20 mM HEPES [pH = 7.4], 1% Triton X-100, 50 mM NaCl, 2 mM EGTA, 5 mM β-glycerophosphate, 30 mM sodium pyrophosphate, 100 mM sodium orthovanadate, 1 mM phenylmethylsulfonyl fluoride, 10 µg/ml aprotinin, 10 µg/ml leupeptin). Insoluble debris was discarded after centrifugation. Whole-cell extracts containing equal amounts of protein (30µg) were separated by SDS-PAGE on 12.5% Laemmli gels, transferred to PVDF membranes presoaked in 100% methanol, and incubated with primary (overnight at 4 °C) and then secondary (one to two hours at room temperature) antibodies. Blots were developed using enhanced chemiluminescence reagents (GE Healthcare) and imaged using a Gel Doc system (BioRad Laboratories, Hercules, CA). Protein expression was quantified by densitometry using Image J software. Results were normalized to the GAPDH loading control and then to the value obtained for untreated control cells to evaluate the fold increase.

### In silico studies

*Protein and ligand structure preparation:* In this study, we used the active structure of LPA1-Gi-protein complex bound to 18:1-LPA (PDB ID: 7TD1). The structure preparation step was done using the MOE software. The *QuickPrep* module in MOE was utilized to generate rotamers, assign appropriate protonation states to residues, add the missing hydrogen atoms, and minimize the structure to remove any bad contacts. All the titratable residues were assigned their dominant protonation states at pH 7.4 using the GBSA solvation model. The 3D atomic coordinates of 1-stearoyl-2-hydroxy-*sn*-glycero-3-phosphate (18:0-LPA) were obtained from PubChem website [22]. The geometries of the ligand structures were further optimized using MOE.

*Molecular Docking:* The molecular docking of 18:0-LPA at the orthosteric binding site of the receptor was done using the docking module in MOE [54]. The binding pocket was defined by select residues, including R124^3.28^, D129^3.33^, W210^5.43^, G274^6.51^, and K294^7.36^ within the orthosteric site of the receptor. We used Alpha PMI and Triangle matcher placement methods to generate multiple poses of the ligands within the receptor. The protein-bound ligand structures were further refined using the induced-fit method, in which the receptor side chains were allowed to be flexible while the backbone was fixed. The docked poses were then ranked using the London dG scoring function. The most appropriate poses were selected based on the docking score, observed interactions, and orientation of the functional groups of the ligand within the orthosteric site.

*Unbiased MD Simulations:* The four receptor-ligand complexes (18:1-LPA-LPA1-Gi protein, 18:1-LPA-LPA1, 18:0-LPA-LPA1-Gi protein, 18:0-LPA-LPA1) were then oriented in the membrane plane using the OPM server [55]. The membrane builder module of the CHARMM-GUI web server was utilized to generate a heterogenous membrane consisting of 16:0/18:1 phosphatidylcholine (POPC) (80–90) and cholesterol (8–11) [56]. The CHARMM36 force field parameters were utilized to model interactions among the system components [57,58]. Ligand parameterization was carried out using CHARMM-GUI ligand reader and modeler, and charges were assigned using CGenFF [59]. The system was solvated with the TIP3P water model padding up to about 23 Å on both sides. The ionic concentration of the final system was at 0.15 M using sufficient NaCl. The final ligand-receptor-membrane complex, which contained 205 lipid molecules, was then subjected to 5000 steps of minimization and thereafter was equilibrated using the CHARMM-GUI recommended six-step equilibration protocol [56,60]. The equilibration steps involve applying various restraints to the system components: 1) harmonic restraints to ions and heavy atoms of the protein, water, ion, and lipid molecules, 2) repulsive planar restraints on water to prevent them from entering into the bilayer core region, and 3) planar restraints to hold the lipid headgroups in position along the bilayer normal. During the initial equilibration step, the harmonic restraints were applied to all the system components at their maximum levels [10, 5, 2.5, 2.5, and 10 kcal/(mol*Å^2^) on protein backbone atoms, protein sidechain, water, lipid and ions, respectively]. In the subsequent steps, the restraints were removed from ions, and the force constants were gradually reduced for other components. Complete details are provided in Table E in S1 Appendix [56,60]. All MD simulations were run using GROMACS 5.1.4 suite [61]. During the production run, van der Waals and short-range electrostatic interactions were estimated with a cut-off distance of 12 Å. The long-range electrostatic interactions were computed using the particle-mesh Ewald summation method. A 2-fs integration time step was used for all MD simulations. The system was simulated under conditions of constant pressure and temperature (NPT conditions) at 1 atm pressure and temperature of 310 K, respectively. The temperature and pressure were controlled using the Nose-Hoover thermostat and the Parinello-Rahman barostat with a coupling constant of 5.0 ps and compressibility of 4.5x10^-5^ bar^-1^. All simulations were carried out for a duration of 500 ns with three replicates per simulation system. Frames from the simulation were saved every 10 picoseconds.

*Clustering the representative pose:* For each ligand, bound-state conformations from all three 500-ns unbiased MD replicates were pooled and clustered (hierarchical, average-linkage) by ligand heavy-atom RMSD (computed relative to binding-site residues) using MDAnalysis [62,63]. The largest occupancy cluster was identified, and its medoid was selected as the “most-preferred binding pose,” a common RMSD-based practice for representative-structure selection in MD clustering. Pose selection was cross-validated against contact-frequency profiles (percent of frames with any heavy-atom pair within 4 Å), ensuring that the displayed pose recapitulates the high-occupancy contacts observed over the trajectories.

*Membrane partitioning simulations:* The membrane partitioning characteristics of 18:0-LPA and 18:1-LPA were investigated using enhanced sampling methods, including steered molecular dynamics (SMD) and umbrella sampling (US), as previously described [64]. Here, membrane partitioning refers to the thermodynamic equilibrium distribution of the ligand between the aqueous phase and the lipid bilayer, quantified as the free energy of transfer along the bilayer normal (ΔG_partitioning_). This is distinct from the ligand access pathway to the receptor, which is addressed separately using WT-MetaD simulations. Ligand parameterization followed the same CHARMM-GUI/CGenFF procedure described above [65]. The membrane was assembled using the CHARMM-GUI Membrane Builder as described above [58,66]. The bilayer composition in both 18:1-LPA and 18:0-LPA simulations included 70 POPC molecules and seven cholesterol molecules in each of the upper and lower leaflets. The system was solvated and ions were added as described above [67]. The CHARMM36 force field parameters were utilized to model interactions among the system components [57,58]. Subsequently, the ligand/bilayer system was then equilibrated following the same six-step CHARMM-GUI protocol described in the Unbiased MD simulations section (see also **Table E** in S1 Appendix), with NVT used for the first two steps, and NPT for the final four. An additional equilibration run was executed for 50 ns before the beginning of the SMD process. At the start of the SMD simulation, the ligand was kept within the aqueous phase at 30 Å from the center of the membrane bilayer. SMD was used to pull the ligand from its position in the aqueous bulk through the membrane bilayer along the bilayer normal (z-axis). The pulling occurred at 1 Å per ns rate, integrated by a one fs time step with a harmonic force restraint of 5 kcal/mol/Å on the LPA molecule. The ligand/bilayer system coordinates at each 1 Å of the permeability path (totaling 30 windows) were obtained from the SMD simulation and used as starting structures for the subsequent umbrella sampling simulations. For the umbrella sampling, the system representing each window was first equilibrated for 10 ns, and a subsequent production simulation was run for an additional 50 ns. This resulted in a cumulative simulation time of ~1.5 µs for each of the ligands studied. The z-component of the distance between the center-of-mass of the lipid atoms and the heavy atoms of the ligand was restrained by a harmonic force of 1.5 kcal/mol/Å during the umbrella sampling simulations. This was implemented through the Colvars module [68]. The obtained probability distributions were reweighted using the weighted histogram analysis method (WHAM) [69] to derive an unbiased Potential Mean Force (PMF). All SMD and US simulations were run using the CUDA version of NAMD 2.12.

#### Ligand association using well-tempered metadynamics (WT-MetaD).

WT-MetaD association simulations [70,71] were carried out in GROMACS 2019 [61,72] patched with PLUMED v2.3 [73,74]. Two collective variables (CVs) were used: (i) CV1 – the center-of-mass (COM) distance between ligand heavy atoms and binding site residues G110^ECL1^, R124^3.28^, W210^5.43^, and E293^7.35^; (ii) CV2 – the ligand internal angle (α) defined by three points: the phosphate head (P1), the middle alkyl segment (C9-10), and the tail end (C1-C2) (Fig 1A). Simulations were run at 310 K with an upper wall of 3.0 nm applied to CV1. WT-MetaD parameters were: Gaussian height = 1.5, σ = 0.05 nm, bias factor = 15, deposition stride = 1 ps, and an additional restraint force of 200 kJ/mol. Each run was terminated when CV1 < 0.5 nm; thus, replicate lengths ranged ~30–50 ns. These association simulations were designed to capture mechanistic entry routes rather than fully converged free-energy estimates. The free-energy surfaces (FES) and minimum free-energy paths were generated with MEPSA, using its connectivity analysis to extract paths [75]. Triplicate runs were performed per ligand for aqueous-phase starts and for starts from membrane-favored locations.

*Network Analysis:* Network analyses of the trajectories from the unbiased simulations were performed to investigate the potential signal transduction paths between the activation site residues (R124, W271, and K294) and the residues that interface with Gi-protein (R152 and K316). In a typical receptor network model, the nodes (represented by amino acid residues) are connected by edges. Two nodes are in contact if their heavy atoms are within 4.5 Å for over 75% of the simulation. The length of a path between two distant nodes is the sum of the edge weights between the consecutive nodes along the path. The shortest path (optimal path) between the distant nodes is assumed to be their most prevalent communication path. The dynamic network analyses between the chosen nodes (residues) were calculated using the *networkView* plugin in VMD software [76–78]. The contact maps between amino acid residues were obtained using the *Carma* software [79]. The communities of closely associated residues were generated using the *gncommunities* module, while the *subopt* program was used to identify suboptimal paths between the residues of interest.

*MM/PBSA Binding Free Energy and Per-Residue Decomposition.* The receptor-ligand binding free energies and per-residue contributions for the two LPAs bound to LPA1 were computed with gmx_MM/PBSA tool [80] on trajectories processed as follows: after 500 ns production, periodicity was removed, trajectories re-centered, and the final 100 ns were analyzed with frames sampled every 1 ns (i.e., 100 frames per system per replicate). The calculation used MM terms (bonded, electrostatics, van der Waals) and a continuum solvation model with Poisson-Boltzmann solvation (igb = 0) [80–82]. The dielectric constants ε_in_= 4.0, ε_out_ = 78.5, and ionic strength = 0.15 M; nonpolar solvation was estimated from solvent-accessible surface area (SASA). Per-residue decomposition employed idecomp = 2 and included residues within 5 Å of the ligand [83]. Protein parameters followed AMBER ff14SB [84–86].

## Supporting information

S1 Appendix The PDF file contains. Physicochemical properties of the two LPA species; MMPBSA binding free energies for the LPA species; Dihedral angle profile of key aromatic residues in experimental LPA1 structures; Membrane partitioning profile of LPA species; CHARMM-GUI six-step equilibration protocol details; Initial binding poses and residue interactions of the LPA species in LPA1; Ligand RMSD and internal angle; Contact fingerprint of the ligands’ glycerophosphate head and alkyl chains with site residues; Time evolution of residue-ligand interactions; binding poses and residue interaction distances in receptor only systems; Comparative analyses of important activation signatures in different LPA1-ligand systems; The chi2 dihedral angle distribution for P308 from NPxxY motif; LPA1-Gi-protein contact maps in the presence of the two LPA species; Water contact analyses; Residue-residue communication paths between orthosteric site residues and activation switches; Replicates of free energy surfaces, characterizing the access and binding of the two LPA species; Membrane partitioning characteristics of the two LPA species; Umbrella sampling histogram overlap from the membrane partitioning simulations; bilayer thickness and area per lipid information from the steered MD and umbrella sampling simulations; lipid-ligand contact maps for the two LPA species at the lowest energy windows from membrane partitioning simulations; Sequence alignment of the six LPA receptors.
(PDF)

S1 Movie Showcases the binding pose and molecular interactions of 18:1 LPA at the orthosteric site of the LPA1 receptor observed in all-atom classical MD simulations. (MP4)

S2 Movie Depicts the binding modes and important residue interactions of 18:0 LPA with the LPA1 receptor observed in all-atom MD simulations. (MP4)

S3 Movie Depicts the interaction between 18:1-LPA-bound LPA1 and the α5 helix of the Gα subunit of Gi-protein. The conserved R146^3.50^ forms a stable hydrogen bond interaction with C351 (from Gαi protein). (MP4)

S4 Movie Depicts the receptor-G-protein interface interaction between 18:0-LPA-bound LPA1 and the α5 helix of the Gα subunit of Gi-protein. (MP4)

S5 Movie Shows the access and binding processes of 18:1 LPA to LPA1 from the extracellular aqueous environment. This association simulation was performed using well-tempered metadynamics.
(MP4)

S6 Movie Illustrates the access and binding processes of 18:0 LPA to the LPA1 receptor from an extracellular aqueous environment. (MP4)

## References

[1] Fukushima N, Ishii I, Contos JJ, Weiner JA, Chun J. Lysophospholipid Receptors. Annu Rev Pharmacol Toxicol. 2001;41:507–34. 11264467 10.1146/annurev.pharmtox.41.1.507

[2] Moolenaar WH, van Meeteren LA, Giepmans BNG. The ins and outs of lysophosphatidic acid signaling. Bioessays. 2004;26(8):870–81. doi: 10.1002/bies.20081 15273989

[3] Hanson MA, Roth CB, Jo E, Griffith MT, Scott FL, Reinhart G, et al. Crystal structure of a lipid G protein-coupled receptor. Science. 2012;335(6070):851–5. doi: 10.1126/science.1215904 22344443 PMC3338336

[4] Choi JW, Herr DR, Noguchi K, Yung YC, Lee C-W, Mutoh T, et al. LPA receptors: subtypes and biological actions. Annu Rev Pharmacol Toxicol. 2010;50:157–86. doi: 10.1146/annurev.pharmtox.010909.105753 20055701

[5] Hla T, Maciag T. An abundant transcript induced in differentiating human endothelial cells encodes a polypeptide with structural similarities to G-protein-coupled receptors. J Biol Chem. 1990;265(16):9308–13. doi: 10.1016/s0021-9258(19)38849-0 2160972

[6] Geraldo LHM, de Sampaio Spohr TCL, do Amaral RF, da Fonseca ACC, Garcia C, Mendes F de A, et al. Role of lysophosphatidic acid and its receptors in health and disease: novel therapeutic strategies. Signal Transduct Target Ther. 2021;6(1):45. doi: 10.1038/s41392-020-00367-5 33526777 PMC7851145

[7] Balijepalli P, Sitton CC, Meier KE. Lysophosphatidic Acid Signaling in Cancer Cells: What Makes LPA So Special? Cells. 2021;10(8):2059. 34440828 10.3390/cells10082059 PMC8394178

[8] Yung YC, Stoddard NC, Chun J. LPA receptor signaling: pharmacology, physiology, and pathophysiology. J Lipid Res. 2014;55(7):1192–214. doi: 10.1194/jlr.R046458 24643338 PMC4076099

[9] Hauser AS, Avet C, Normand C, Mancini A, Inoue A, Bouvier M, et al. Common coupling map advances GPCR-G protein selectivity. eLife. 2022;11:e74107. doi: 10.7554/eLife.74107 35302494 PMC9005189

[10] Chaudhary PK, Kim S. An Insight into GPCR and G-Proteins as Cancer Drivers. Cells. 2021;10(12):3288. doi: 10.3390/cells10123288 34943797 PMC8699078

[11] Sattikar A, Dowling MR, Rosethorne EM. Endogenous lysophosphatidic acid (LPA1) receptor agonists demonstrate ligand bias between calcium and ERK signalling pathways in human lung fibroblasts. Br J Pharmacol. 2017;174(3):227–37. doi: 10.1111/bph.13671 27864940 PMC5241385

[12] Jesionowska A, Cecerska E, Dolegowska B. Methods for quantifying lysophosphatidic acid in body fluids: a review. Anal Biochem. 2014;453:38–43. doi: 10.1016/j.ab.2014.02.021 24613261

[13] Blaho VA, Chun J. “Crystal” Clear? Lysophospholipid Receptor Structure Insights and Controversies. Trends Pharmacol Sci. 2018;39(11):953–66. doi: 10.1016/j.tips.2018.08.006 30343728 PMC6201317

[14] Baker DL, Umstot ES, Desiderio DM, Tigyi GJ. Quantitative analysis of lysophosphatidic acid in human blood fractions. Ann N Y Acad Sci. 2000;905:267–9. doi: 10.1111/j.1749-6632.2000.tb06557.x 10818461

[15] García-Marchena N, Pizarro N, Pavón FJ, Martínez-Huélamo M, Flores-López M, Requena-Ocaña N, et al. Potential association of plasma lysophosphatidic acid (LPA) species with cognitive impairment in abstinent alcohol use disorders outpatients. Sci Rep. 2020;10(1):17163. doi: 10.1038/s41598-020-74155-0 33051508 PMC7555527

[16] Bandoh K, Aoki J, Taira A, Tsujimoto M, Arai H, Inoue K. Lysophosphatidic acid (LPA) receptors of the EDG family are differentially activated by LPA species. FEBS Lett. 2000;478(1–2):159–65. doi: 10.1016/s0014-5793(00)01827-5 10922489

[17] Xie Y, Gibbs TC, Mukhin YV, Meier KE. Role for 18:1 lysophosphatidic acid as an autocrine mediator in prostate cancer cells. J Biol Chem. 2002;277(36):32516–26. doi: 10.1074/jbc.M203864200 12084719

[18] Hopkins MM, Liu Z, Meier KE. Positive and Negative Cross-Talk between Lysophosphatidic Acid Receptor 1, Free Fatty Acid Receptor 4, and Epidermal Growth Factor Receptor in Human Prostate Cancer Cells. J Pharmacol Exp Ther. 2016;359(1):124–33. doi: 10.1124/jpet.116.233379 27474750 PMC5034703

[19] Suenaga R, Takemoto M, Inoue A, Ishitani R, Nureki O. Lateral access mechanism of LPA receptor probed by molecular dynamics simulation. PLoS One. 2022;17(2):e0263296. doi: 10.1371/journal.pone.0263296 35113924 PMC8812926

[20] Salgado-Polo F, Borza R, Matsoukas M-T, Marsais F, Jagerschmidt C, Waeckel L, et al. Autotaxin facilitates selective LPA receptor signaling. Cell Chem Biol. 2023;30(1):69–84.e14. doi: 10.1016/j.chembiol.2022.12.006 36640760

[21] Valdés-Rives SA, González-Arenas A. Autotaxin-lysophosphatidic acid: from inflammation to cancer development. Mediators Inflamm. 2017;9173090. doi: 10.1155/2017/9173090 PMC5753009 29430083

[22] Kim S, Chen J, Cheng T, Gindulyte A, He J, He S, et al. PubChem in 2021: new data content and improved web interfaces. Nucl Acids Res. 2020;49:D1388–95. 10.1093/nar/gkaa971 PMC7778930 33151290

[23] Leekumjorn S, Cho HJ, Wu Y, Wright NT, Sum AK, Chan C. The role of fatty acid unsaturation in minimizing biophysical changes on the structure and local effects of bilayer membranes. Biochim Biophys Acta. 2009;1788(7):1508–16. doi: 10.1016/j.bbamem.2009.04.002 19371719 PMC2698950

[24] Wong-Ekkabut J, Xu Z, Triampo W, Tang I-M, Tieleman DP, Monticelli L. Effect of lipid peroxidation on the properties of lipid bilayers: a molecular dynamics study. Biophys J. 2007;93(12):4225–36. doi: 10.1529/biophysj.107.112565 17766354 PMC2098729

[25] Troupiotis-Tsaïlaki A, Zachmann J, González-Gil I, Gonzalez A, Ortega-Gutiérrez S, López-Rodríguez ML, et al. Ligand chain length drives activation of lipid G protein-coupled receptors. Sci Rep. 2017;7(1):2020. doi: 10.1038/s41598-017-02104-5 28515494 PMC5435731

[26] Chrencik JE, Roth CB, Terakado M, Kurata H, Omi R, Kihara Y, et al. Crystal Structure of Antagonist Bound Human Lysophosphatidic Acid Receptor 1. Cell. 2015;161(7):1633–43. doi: 10.1016/j.cell.2015.06.002 26091040 PMC4476059

[27] Liu S, Paknejad N, Zhu L, Kihara Y, Ray M, Chun J, et al. Differential activation mechanisms of lipid GPCRs by lysophosphatidic acid and sphingosine 1-phosphate. Nat Commun. 2022;13(1):731. doi: 10.1038/s41467-022-28417-2 35136060 PMC8826421

[28] Akasaka H, Tanaka T, Sano FK, Matsuzaki Y, Shihoya W, Nureki O. Structure of the active Gi-coupled human lysophosphatidic acid receptor 1 complexed with a potent agonist. Nat Commun. 2022;13(1):5417. doi: 10.1038/s41467-022-33121-2 36109516 PMC9477835

[29] Akasaka H, Sano FK, Shihoya W, Nureki O. Structural mechanisms of potent lysophosphatidic acid receptor 1 activation by nonlipid basic agonists. Commun Biol. 2024;7(1):1444. doi: 10.1038/s42003-024-07152-y 39506093 PMC11541586

[30] Zhou Q, Yang D, Wu M, Guo Y, Guo W, Zhong L, et al. Common activation mechanism of class A GPCRs. eLife. 2019;8:e50279. 10.7554/eLife.50279 PMC6954041 31855179

[31] Wingert B, Doruker P, Bahar I. Activation and Speciation Mechanisms in Class A GPCRs. J Mol Biol. 2022;434(17):167690. doi: 10.1016/j.jmb.2022.167690 35728652 PMC10129049

[32] Mafi A, Kim SK, Goddard WA. The mechanism for ligand activation of the GPCR–G protein complex. Proc Natl Acad Sci U S A. 2023;119:e2110085119. 10.1073/pnas.2110085119 PMC9170043 35452328

[33] Yang D, Zhou Q, Labroska V, Qin S, Darbalaei S, Wu Y, et al. G protein-coupled receptors: structure- and function-based drug discovery. Signal Transduct Target Ther. 2021;6(7). 10.1038/s41392-020-00435-w PMC7790836 33414387

[34] Bokoch MP, Jo H, Valcourt JR, Srinivasan Y, Pan AC, Capponi S, et al. Entry from the Lipid Bilayer: A Possible Pathway for Inhibition of a Peptide G Protein-Coupled Receptor by a Lipophilic Small Molecule. Biochemistry. 2018;57(39):5748–58. doi: 10.1021/acs.biochem.8b00577 30102523 PMC6584023

[35] Hurst DP, Grossfield A, Lynch DL, Feller S, Romo TD, Gawrisch K, et al. A lipid pathway for ligand binding is necessary for a cannabinoid G protein-coupled receptor. J Biol Chem. 2010;285(23):17954–64. doi: 10.1074/jbc.M109.041590 20220143 PMC2878557

[36] Stanley N, Pardo L, Fabritiis GD. The pathway of ligand entry from the membrane bilayer to a lipid G protein-coupled receptor. Sci Rep. 2016;6:22639. doi: 10.1038/srep22639 26940769 PMC4778059

[37] Dror RO, Pan AC, Arlow DH, Borhani DW, Maragakis P, Shan Y, et al. Pathway and mechanism of drug binding to G-protein-coupled receptors. Proc Natl Acad Sci U S A. 2011;108(32):13118–23. doi: 10.1073/pnas.1104614108 21778406 PMC3156183

[38] Jakowiecki J, Orzeł U, Chawananon S, Miszta P, Filipek S. The Hydrophobic Ligands Entry and Exit from the GPCR Binding Site-SMD and SuMD Simulations. Molecules. 2020;25(8):1930. doi: 10.3390/molecules25081930 32326322 PMC7221835

[39] Milligan G, Shimpukade B, Ulven T, Hudson BD. Complex pharmacology of free fatty acid receptors. Chem Rev. 2017;117:67–110. 27299848 10.1021/acs.chemrev.6b00056

[40] Liu Z, Hopkins MM, Zhang Z, Quisenberry CB, Fix LC, Galvan BM, et al. Omega-3 fatty acids and other FFA4 agonists inhibit growth factor signaling in human prostate cancer cells. J Pharmacol Exp Ther. 2015;352(2):380–94. doi: 10.1124/jpet.114.218974 25491146 PMC4293432

[41] Gibbs TC, Rubio MV, Zhang Z, Xie Y, Kipp KR, Meier KE. Signal transduction responses to lysophosphatidic acid and sphingosine 1-phosphate in human prostate cancer cells. Prostate. 2009;69(14):1493–506. doi: 10.1002/pros.20994 19536794

[42] Balijepalli P, Knode BK, Nahulu SA, Abrahamson EL, Nivison MP, Meier KE. Role for CCN1 in lysophosphatidic acid response in PC-3 human prostate cancer cells. J Cell Commun Signal. 2024;18(1):e12019. doi: 10.1002/ccs3.12019 38545253 PMC10964937

[43] Valentine WJ, Fells JI, Perygin DH, Mujahid S, Yokoyama K, Fujiwara Y, et al. Subtype-specific residues involved in ligand activation of the endothelial differentiation gene family lysophosphatidic acid receptors. J Biol Chem. 2008;283(18):12175–87. doi: 10.1074/jbc.M708847200 18316373 PMC3774115

[44] Manglik A, Kruse AC. Structural basis for G protein-coupled receptor activation. Biochemistry. 2017;56:5628–34. 28967738 10.1021/acs.biochem.7b00747 PMC6613644

[45] Hauser AS, Kooistra AJ, Munk C, Heydenreich FM, Veprintsev DB, Bouvier M, et al. GPCR activation mechanisms across classes and macro/microscales. Nat Struct Mol Biol. 2021;28(11):879–88. doi: 10.1038/s41594-021-00674-7 34759375 PMC8580822

[46] Filipek S. Molecular switches in GPCRs. Curr Opin Struct Biol. 2019;55:114–20. 31082695 10.1016/j.sbi.2019.03.017

[47] Venkatakrishnan AJ, Ma AK, Fonseca R, Latorraca NR, Kelly B, Betz RM, et al. Diverse GPCRs exhibit conserved water networks for stabilization and activation. Proc Natl Acad Sci U S A. 2019;116(8):3288–93. doi: 10.1073/pnas.1809251116 30728297 PMC6386714

[48] Yuan S, Filipek S, Palczewski K, Vogel H. Activation of G-protein-coupled receptors correlates with the formation of a continuous internal water pathway. Nat Commun. 2014;5:4733. doi: 10.1038/ncomms5733 25203160

[49] Ricarte A, Dalton JAR, Giraldo J. Structural Assessment of Agonist Efficacy in the μ-Opioid Receptor: Morphine and Fentanyl Elicit Different Activation Patterns. J Chem Inf Model. 2021;61(3):1251–74. doi: 10.1021/acs.jcim.0c00890 33448226

[50] Omotuyi OI, Adebowale DD, Famuti A, Tsuyoshi H. LPA1extracellular loop residues 115 and 191 are not required for receptor activation but prevent Ki16425 super-antagonism. RSC Adv. 2016;6(60):55257–65. doi: 10.1039/c6ra04276g

[51] Jiang Z, Zhang H. Molecular Mechanism of S1P Binding and Activation of the S1P1 Receptor. J Chem Inf Model. 2019;59(10):4402–12. doi: 10.1021/acs.jcim.9b00642 31589433

[52] Szlenk CT, Gc JB, Natesan S. Does the Lipid Bilayer Orchestrate Access and Binding of Ligands to Transmembrane Orthosteric/Allosteric Sites of G Protein-Coupled Receptors? Mol Pharmacol. 2019;96(5):527–41. doi: 10.1124/mol.118.115113 30967440 PMC6776015

[53] Ghanouni P, Gryczynski Z, Steenhuis JJ, Lee TW, Farrens DL, Lakowicz JR, et al. Functionally different agonists induce distinct conformations in the G protein coupling domain of the beta 2 adrenergic receptor. J Biol Chem. 2001;276(27):24433–6. doi: 10.1074/jbc.C100162200 11320077

[54] Molecular Operating Environment (MOE). 2024.0641 Chemical Computing Group ULC, 910-1010 Sherbrooke St. W., Montreal, QC, Canada H3A 2R7. 2026.

[55] Lomize MA, Pogozheva ID, Joo H, Mosberg HI, Lomize AL. OPM database and PPM web server: resources for positioning of proteins in membranes. Nucleic Acids Res. 2012;40(Database issue):D370-6. doi: 10.1093/nar/gkr703 21890895 PMC3245162

[56] Jo S, Lim JB, Klauda JB, Im W. CHARMM-GUI Membrane Builder for mixed bilayers and its application to yeast membranes. Biophys J. 2009;97(1):50–8. doi: 10.1016/j.bpj.2009.04.013 19580743 PMC2711372

[57] Klauda JB, Venable RM, Freites JA, O’Connor JW, Tobias DJ, Mondragon-Ramirez C, et al. Update of the CHARMM all-atom additive force field for lipids: validation on six lipid types. J Phys Chem B. 2010;114(23):7830–43. doi: 10.1021/jp101759q 20496934 PMC2922408

[58] Lee J, Cheng X, Swails JM, Yeom MS, Eastman PK, Lemkul JA, et al. CHARMM-GUI Input Generator for NAMD, GROMACS, AMBER, OpenMM, and CHARMM/OpenMM Simulations Using the CHARMM36 Additive Force Field. J Chem Theory Comput. 2016;12(1):405–13. doi: 10.1021/acs.jctc.5b00935 26631602 PMC4712441

[59] Vanommeslaeghe K, Hatcher E, Acharya C, Kundu S, Zhong S, Shim J, et al. CHARMM general force field: A force field for drug-like molecules compatible with the CHARMM all-atom additive biological force fields. J Comput Chem. 2010;31(4):671–90. doi: 10.1002/jcc.21367 19575467 PMC2888302

[60] Jo S, Kim T, Im W. Automated builder and database of protein/membrane complexes for molecular dynamics simulations. PLoS ONE. 2007;2:e880. 10.1371/journal.pone.0000880 PMC1963319 17849009

[61] Abraham MJ, Murtola T, Schulz R, Páll S, Smith JC, Hess B, et al. GROMACS: High performance molecular simulations through multi-level parallelism from laptops to supercomputers. SoftwareX. 2015;1–2:19–25. doi: 10.1016/j.softx.2015.06.001

[62] Michaud-Agrawal N, Denning EJ, Woolf TB, Beckstein O. MDAnalysis: a toolkit for the analysis of molecular dynamics simulations. J Comput Chem. 2011;32(10):2319–27. doi: 10.1002/jcc.21787 21500218 PMC3144279

[63] Gowers R, Linke M, Barnoud J, Reddy T, Melo M, Seyler S, et al. MDAnalysis: A Python Package for the Rapid Analysis of Molecular Dynamics Simulations. Proceedings of the 15th Python in Science Conference, 2016. 2016. p. 98–105.

[64] Obi P, Gc JB, Mariasoosai C, Diyaolu A, Natesan S. Application of Generative Artificial Intelligence in Predicting Membrane Partitioning of Drugs: Combining Denoising Diffusion Probabilistic Models and MD Simulations Reduces the Computational Cost to One-Third. J Chem Theory Comput. 2024;20(14):5866–81. doi: 10.1021/acs.jctc.4c00315 38942732

[65] Brooks BR, Brooks CL 3rd, Mackerell AD Jr, Nilsson L, Petrella RJ, Roux B, et al. CHARMM: the biomolecular simulation program. J Comput Chem. 2009;30(10):1545–614. doi: 10.1002/jcc.21287 19444816 PMC2810661

[66] Wu EL, Cheng X, Jo S, Rui H, Song KC, Dávila-Contreras EM, et al. CHARMM-GUI Membrane Builder toward realistic biological membrane simulations. J Comput Chem. 2014;35(27):1997–2004. doi: 10.1002/jcc.23702 25130509 PMC4165794

[67] Jorgensen WL, Chandrasekhar J, Madura JD, Impey RW, Klein ML. Comparison of simple potential functions for simulating liquid water. J Chem Phys. 1983;79:926–35.

[68] Fiorin G, Klein ML, Hénin J. Using collective variables to drive molecular dynamics simulations. Molecular Physics. 2013;111(22–23):3345–62. doi: 10.1080/00268976.2013.813594

[69] Kumar S, Rosenberg JM, Bouzida D, Swendsen RH, Kollman PA. The weighted histogram analysis method for free-energy calculations on biomolecules. I. The method. J Comput Chem. 1992;13:1011–21.

[70] Barducci A, Bussi G, Parrinello M. Well-tempered metadynamics: A smoothly converging and tunable free-energy method. Phys Rev Lett. 2008;100:020603. 18232845 10.1103/PhysRevLett.100.020603

[71] Limongelli V, Bonomi M, Parrinello M. Funnel metadynamics as accurate binding free-energy method. Proc Natl Acad Sci U S A. 2013;110(16):6358–63. doi: 10.1073/pnas.1303186110 23553839 PMC3631651

[72] Berendsen HJC, van der Spoel D, van Drunen R. GROMACS: A message-passing parallel molecular dynamics implementation. Comput Phys Commun. 1995;91:43–56.

[73] Bonomi M, Branduardi D, Bussi G, Camilloni C, Provasi D, Raiteri P, et al. PLUMED: A portable plugin for free-energy calculations with molecular dynamics. Comput Phys Commun. 2009;180:1961–72.

[74] Tribello GA, Bonomi M, Branduardi D, Camilloni C, Bussi G. PLUMED 2: New feathers for an old bird. Comput Phys Commun. 2014;185:604–13.

[75] Marcos-Alcalde I, Setoain J, Mendieta-Moreno JI, Mendieta J, Gómez-Puertas P. MEPSA: minimum energy pathway analysis for energy landscapes. Bioinformatics. 2015;31(23):3853–5. doi: 10.1093/bioinformatics/btv453 26231428

[76] Eargle J, Luthey-Schulten Z. NetworkView: 3D display and analysis of protein·RNA interaction networks. Bioinformatics. 2012;28(22):3000–1. doi: 10.1093/bioinformatics/bts546 22982572 PMC3496333

[77] Humphrey W, Dalke A, Schulten K. VMD: visual molecular dynamics. J Mol Graph. 1996;14(1):33–8, 27–8. doi: 10.1016/0263-7855(96)00018-5 8744570

[78] Gc JB, Szlenk CT, Diyaolu A, Obi P, Wei H, Shi X, et al. Allosteric modulation of α1β3γ2 GABAA receptors by farnesol through the neurosteroid sites. Biophys J. 2023;122(5):849–67. doi: 10.1016/j.bpj.2023.01.032 36721367 PMC10027449

[79] Glykos NM. Software news and updates. Carma: a molecular dynamics analysis program. J Comput Chem. 2006;27(14):1765–8. doi: 10.1002/jcc.20482 16917862

[80] Valdés-Tresanco MS, Valdés-Tresanco ME, Valiente PA, Moreno E. gmx_MMPBSA: A New Tool to Perform End-State Free Energy Calculations with GROMACS. J Chem Theory Comput. 2021;17(10):6281–91. doi: 10.1021/acs.jctc.1c00645 34586825

[81] Kollman PA, Massova I, Reyes C, Kuhn B, Huo S, Chong L, et al. Calculating Structures and Free Energies of Complex Molecules: Combining Molecular Mechanics and Continuum Models. Acc Chem Res. 2000;33:889–97. 11123888 10.1021/ar000033j

[82] Srinivasan J, Cheatham TE, Cieplak P, Kollman PA, Case DA. Continuum solvent studies of the stability of DNA, RNA, and phosphoramidate−DNA helices. J Am Chem Soc. 1998;120:9401–9.

[83] Birch J, Cheruvara HA-O, Gamage N, Harrison PA-O, Lithgo RA-O, Quigley A. Changes in Membrane Protein Structural Biology. Biology (Basel). 2020;9(11):401. 33207666 10.3390/biology9110401 PMC7696871

[84] Perez A, Morrone JA, Simmerling C, Dill KA. Advances in free-energy-based simulations of protein folding and ligand binding. Curr Opin Struct Biol. 2016;36:25–31. doi: 10.1016/j.sbi.2015.12.002 26773233 PMC4785060

[85] Hornak V, Abel R, Okur A, Strockbine B, Roitberg A, Simmerling C. Comparison of multiple Amber force fields and development of improved protein backbone parameters. Proteins. 2006;65(3):712–25. doi: 10.1002/prot.21123 16981200 PMC4805110

[86] Cheng X, Hornak V, Simmerling C. Improved conformational sampling through an efficient combination of mean-field simulation approaches. J Phys Chem B. 2004;108:426–37.

