# Supplementary material for: Structural mechanisms underlying distinct binding and activities of 18:0 and 18:1 lysophosphatidic acids at LPA1 receptor
Source: PLoS Comput Biol. 2026 Apr 24;22(4):e1013825. doi: 10.1371/journal.pcbi.1013825 (PMC13132450; doi:10.1371/journal.pcbi.1013825)
Supplement: S1 Appendix — Physicochemical properties of the two LPA species; MMPBSA binding free energies for the LPA species; Dihedral angle profile of key aromatic residues in experimental LPA1 structures; Membrane partitioning profile of LPA species; CHARMM-GUI six-step equilibration protocol details; Initial binding poses and residue interactions of the LPA species in LPA1; Ligand RMSD and internal angle; Contact fingerprint of the ligands’ glycerophosphate head and alkyl chains with site residues; Time evolution of residue-ligand interactions; binding poses and residue interaction distances in receptor only systems; Comparative analyses of important activation signatures in different LPA1-ligand systems; The chi2 dihedral angle distribution for P308 from NPxxY motif; LPA1-Gi-protein contact maps in the presence of the two LPA species; Water contact analyses; Residue-residue communication paths between orthosteric site residues and activation switches; Replicates of free energy surfaces, characterizing the access and binding of the two LPA species; Membrane partitioning characteristics of the two LPA species; Umbrella sampling histogram overlap from the membrane partitioning simulations; bilayer thickness and area per lipid information from the steered MD and umbrella sampling simulations; lipid-ligand contact maps for the two LPA species at the lowest energy windows from membrane partitioning simulations; Sequence alignment of the six LPA receptors. (PDF) [file pcbi.1013825.s001.pdf]

# Supporting Information

## Structural Mechanisms Underlying Distinct Binding and Activities of 18:0 and 18:1 Lysophosphatidic Acids at LPA1 Receptor

Ayobami Diyaolu, Peter Obi, Pravita Balijepalli, Kathryn Meier* and Senthil Natesan*

*College of Pharmacy and Pharmaceutical Sciences, Washington State University, Spokane, WA 99202, USA*

**Table A. Physicochemical properties of 18:0-LPA and 18:1-LPA species**

| Physicochemical property | 18:0-LPA | 18:1-LPA |
|---|---|---|
| Molecular weight (g/mol) | 438.5 | 436.5 |
| ClogP | 5.85 | 5.49 |
| Water solubility (g/L) | 0.0015 | 0.0016 |
| Number of rotatable bonds | 22 | 21 |
| Number of hydrogen bond acceptors | 5 | 5 |
| Number of hydrogen bond donors | 3 | 3 |
| Heavy atom count | 29 | 29 |
| TPSA ($Å^2$) | 113.29 | 113.29 |
| Polarizability ($Å^3$) | 51.46 | 49.95 |
| pKa | 1.51 | 1.51 |
| Complexity[†] | 425 | 462 |

†- The complexity rating estimates how complicated a chemical compound is based on its elements and structural features, including symmetry. It is computed using the Bertz/Hendrickson/Ihlenfeldt formula.

**Table B. The binding free energy profiles for the two LPA species were calculated using the MM/PBSA method. All the energy terms reflect the deltaG (Complex – Receptor - ligand) values**

| Energy Component | Binding energy (kcal/mol) | |
|---|---|---|
| | **18:0-LPA** | **18:1-LPA** |
| van der Waals | -44.6 ± 5.16 | -45.2 ± 4.43 |
| electrostatic | -120.6 ± 4.65 | -121.9 ± 5.3 |
| PB | 108.9 ± 3.83 | 112.4 ± 3.96 |
| Surf | -9.32 ± 0.40 | -9.2 ± 0.31 |
| Gas | -165.15 ± 5.68 | -167.1 ± 6.36 |
| Solvation | 99.6 ± 3.82 | 103.2 ± 3.94 |
| Total | **-65.6 ± 4.21** | **-63.9 ± 4.59** |

**Table C. Chi2 dihedral angle for various residues in experimental structures of the LPA1 receptor**

| Residue | chi2 dihedral angle (degree) | | | |
|---|---|---|---|---|
| | Active state LPA1 | | Inactive state LPA1 | |
| | **7TD0** | **7TD1** | **4Z34** | **4Z35** |
| W210 | 84.1 | 92.0 | -99.7 | -100.1 |
| F267 | -72.1 | -76.8 | 91.0 | 91.1 |
| W271 | 108.6 | 99.3 | 102.3 | 101.1 |
| P308 | -32.9 | -32.3 | 34.3 | 35.8 |
| Y311 | 79.5 | 85.2 | -62.0 | -61.7 |

**Table D. The membrane partitioning/crossing profiles and preferred bilayer locations of 18:0-LPA and 18:1-LPA**

| **Ligand** | **$G_{partitioning}$** (kcal/mol) | **$G_{crossing}$** (kcal/mol) | **Energy minimum position of COM of the ligand $z_{min}$ (Å)** | **Thermally accessible region (RT = 0.616 kcal/mol) z (Å)** |
|---|---|---|---|---|
| 18:0 LPA | -9.30 ± 0.05 | 11.06 ± 0.06 | 15 | 13-18 |
| 18:1 LPA | -7.24 ± 0.07 | 12.36 ± 0.08 | 15 | 13-18 |

**Table E. Details of the CHARMM-GUI six step equilibration protocol**

| **Step** | **Ensemble** | **Timesteps (fs)** | **Equilibration time (ps)** | **Force constants for harmonic restraint (kcal/mol*Å$^2$)** | | | | |
|---|---|---|---|---|---|---|---|---|
| | | | | **protein backbone** | **protein sidechain** | **water** | **lipid** | **ions** |
| 1 | NVT | 1 | 25 | 10.0 | 5.0 | 2.5 | 2.5 | 10.0 |
| 2 | NVT | 1 | 25 | 5.0 | 2.5 | 2.5 | 2.5 | 0.0 |
| 3 | NPAT | 1 | 25 | 2.5 | 1.0 | 1.0 | 1.0 | 0.0 |
| 4 | NPAT | 2 | 100 | 1.0 | 0.5 | 0.5 | 0.5 | 0.0 |
| 5 | NPAT | 2 | 100 | 0.5 | 0.1 | 0.1 | 0.1 | 0.0 |
| 6 | NPAT | 2 | 100 | 0.1 | 0.0 | 0.0 | 0.0 | 0.0 |

*NVT – constant volume and temperature; NPAT – constant pressure, area, and temperature

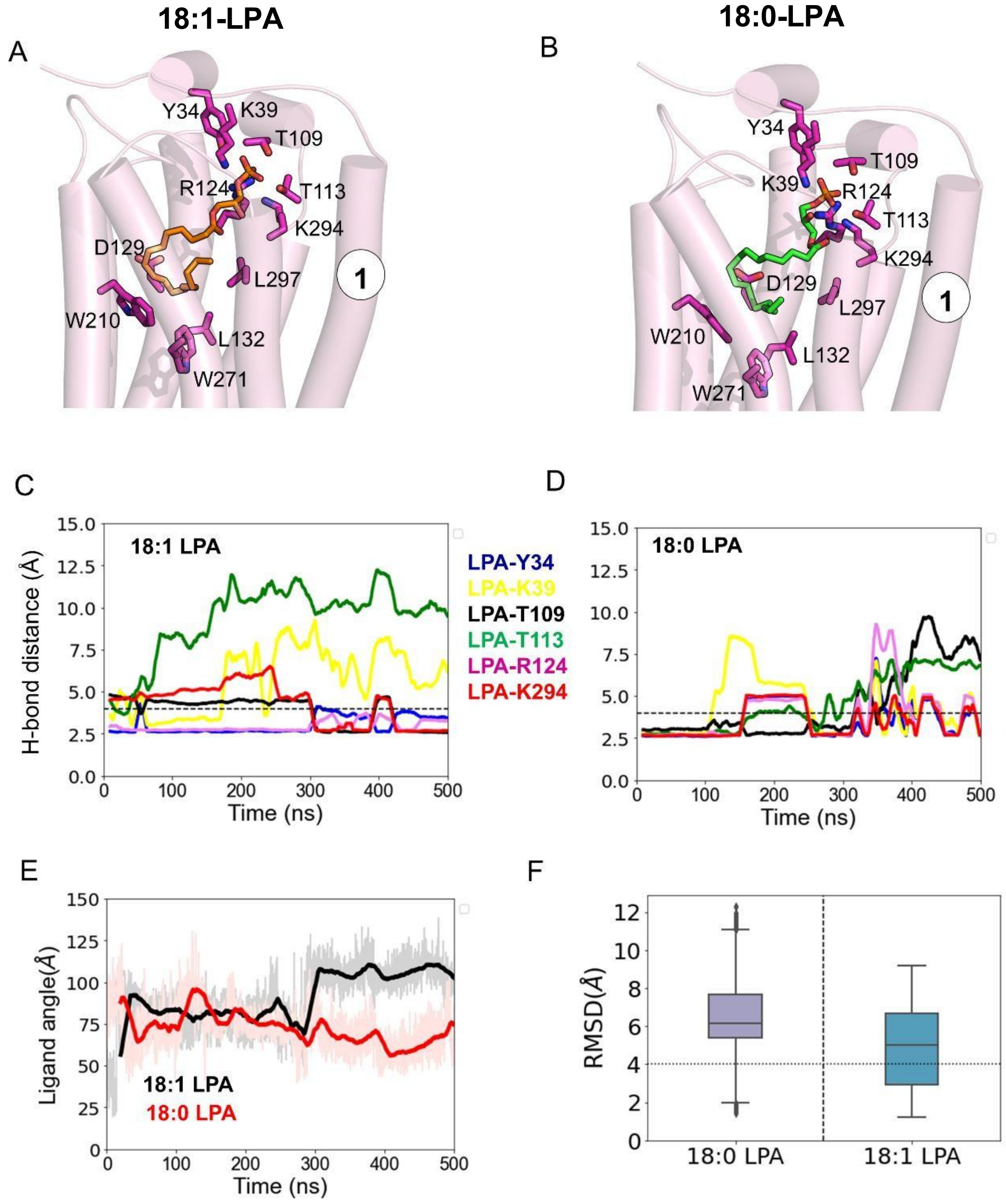


**Figure A. The starting poses, interactions, orientations, and stability of the LPA species bound to the LPA1-Gi protein complex**. (A) A cartoon representation of the cryo-EM structure of LPA1 (PDB ID: 7TD1) with co-crystallized 18:1 LPA (orange licorice). (B) The selected starting pose of 18:0 LPA (green licorice representation) in LPA1 was obtained via docking simulations. (C) The time-based evolution of polar interactions between 18:1 LPA's glycerophosphate group and important orthosteric site residues. (D) The time-based evolution of polar interactions between 18:0 LPA and site residues. (E) The internal angle α of the LPA species (as defined in Results and Methods sections) is shown for 18:0 LPA (red) and 18:1 LPA (black). (F) The boxplots show the distribution of root-mean-square deviations (RMSDs) of 18:0 LPA and 18:1 LPA from 500 ns simulation data

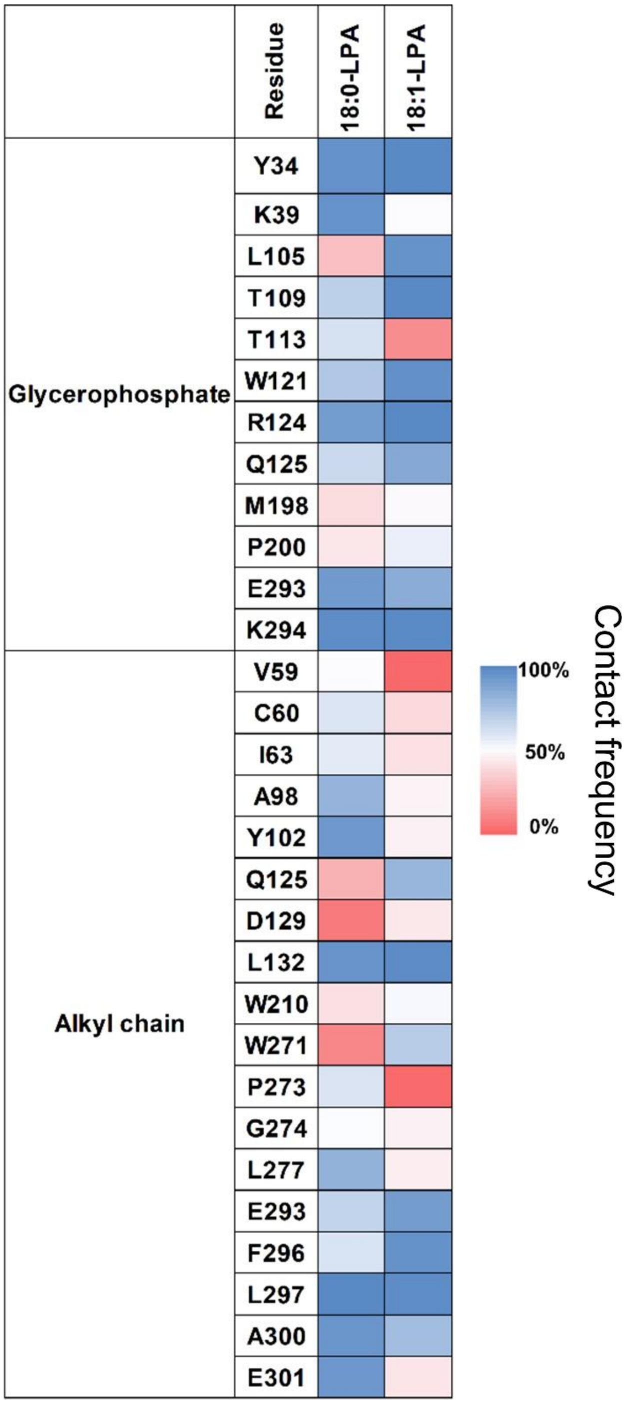


**Figure B. LPA1 makes contact with both polar and hydrophobic parts of the ligands.** (A) The heatmap depicts the percentage contact frequency of the glycerophosphate head and alkyl chain of the ligands with the LPA1 binding site residues. The contact frequency refers to the percentage of the simulation time during which a given residue is within 4 Å of the ligand.

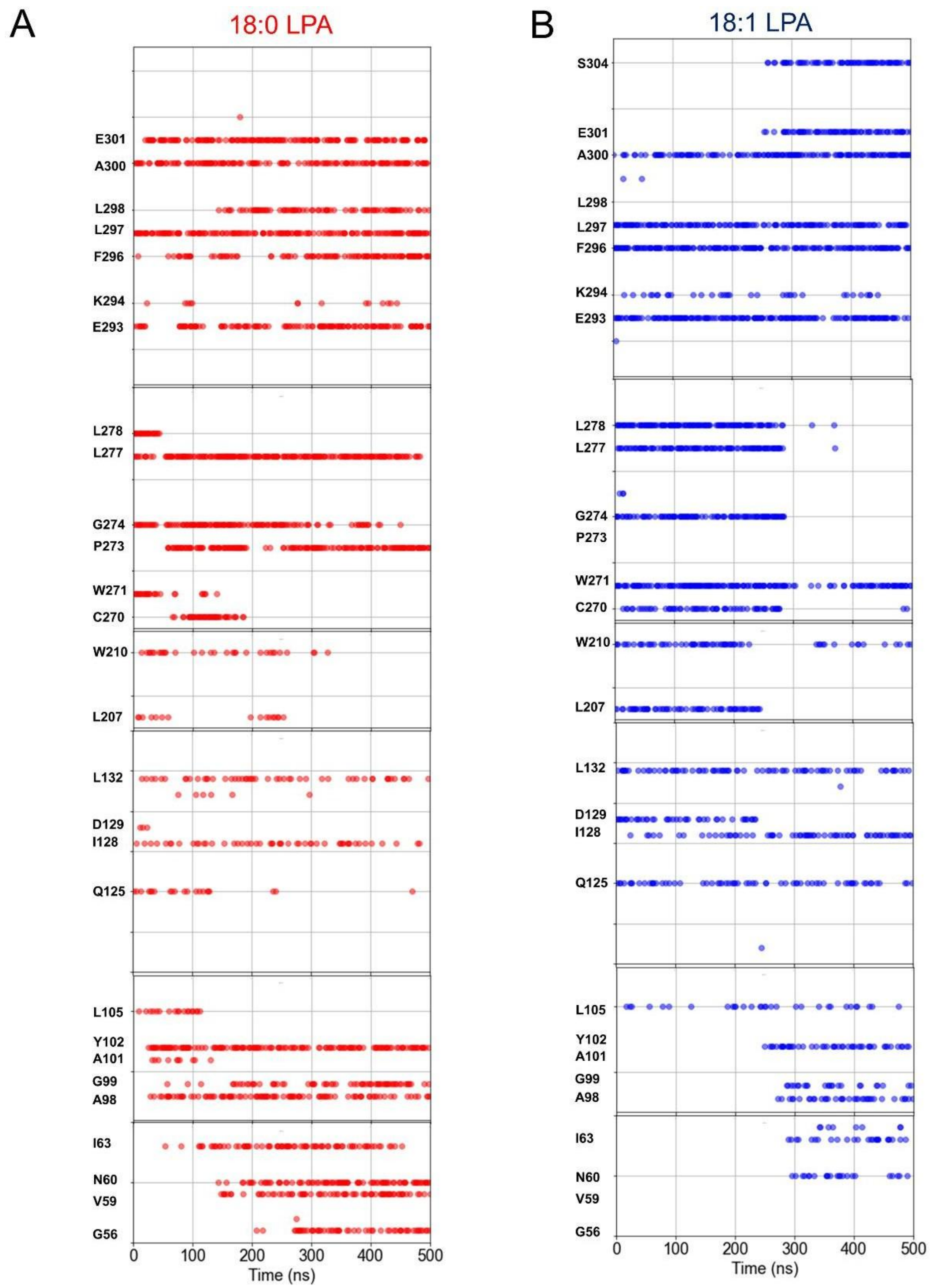


**Figure C. Evolution of the two LPA species' alkyl chain contacts with binding site residues of LPA1 during the simulation**. (A) The time course of the contact of the 18:0 LPA alkyl (C1-C17) chain with residues of LPA1. (B). The time course of 18:1 LPA alkyl chain (C1-C17) contact with residues of LPA1.

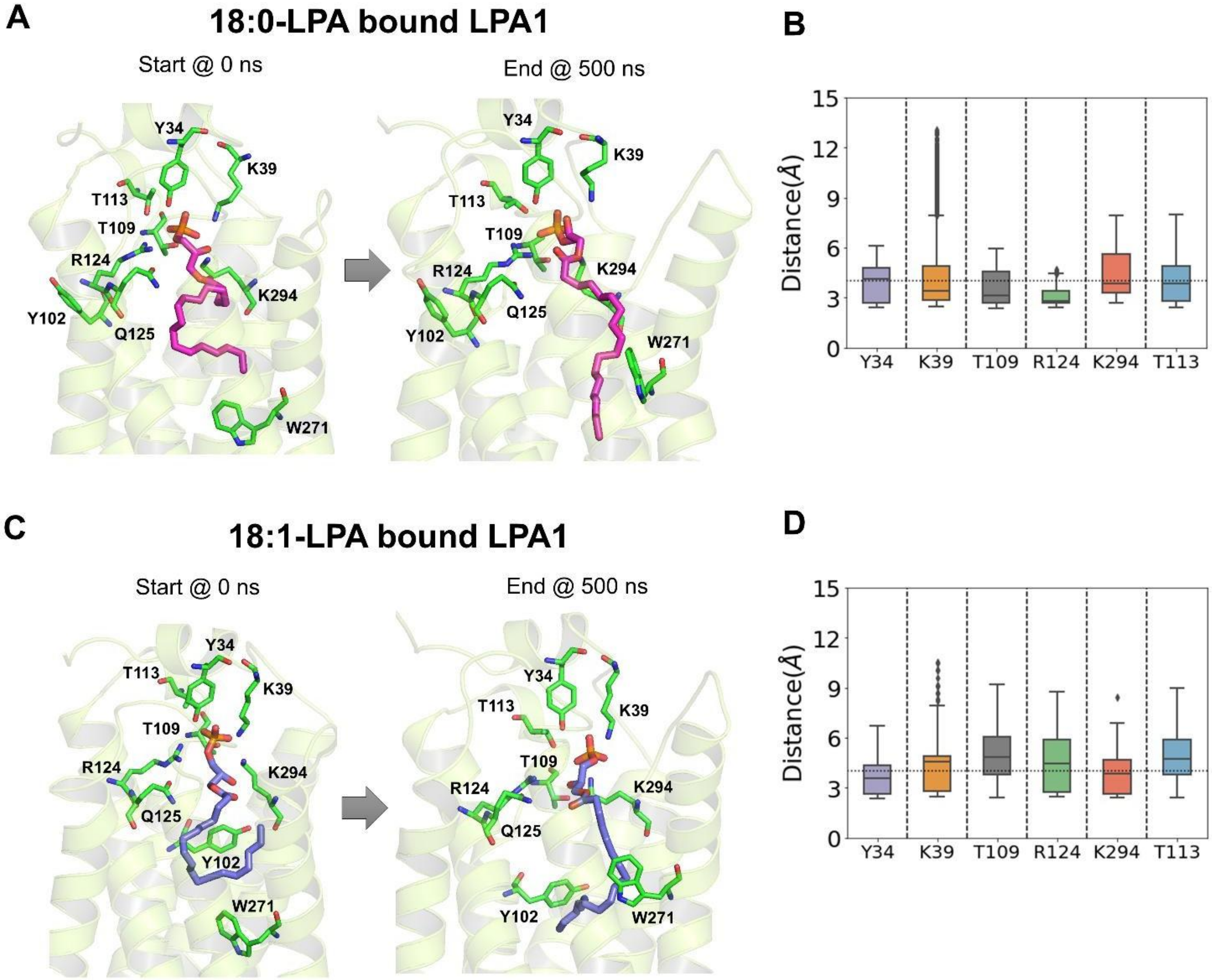


**Figure D. The binding poses of the LPA species in receptor-only systems before and after classical MD simulations.** (A). Conformational changes in the binding orientation of 18:0 LPA (purple licorice) within the orthosteric binding pocket before and after 500 ns simulation. (B) The binding site residues make stable hydrogen bonds and salt bridge interactions with the glycerol-phosphate head of 18:0 LPA. (C) Conformational changes in the binding pose of 18:1 LPA (shown in blue) within the orthosteric binding pocket before and after the simulation. (D) 18:1 LPA establishes strong polar interactions with Y34, T109, R124, and K294 through its glycerol-phosphate group.

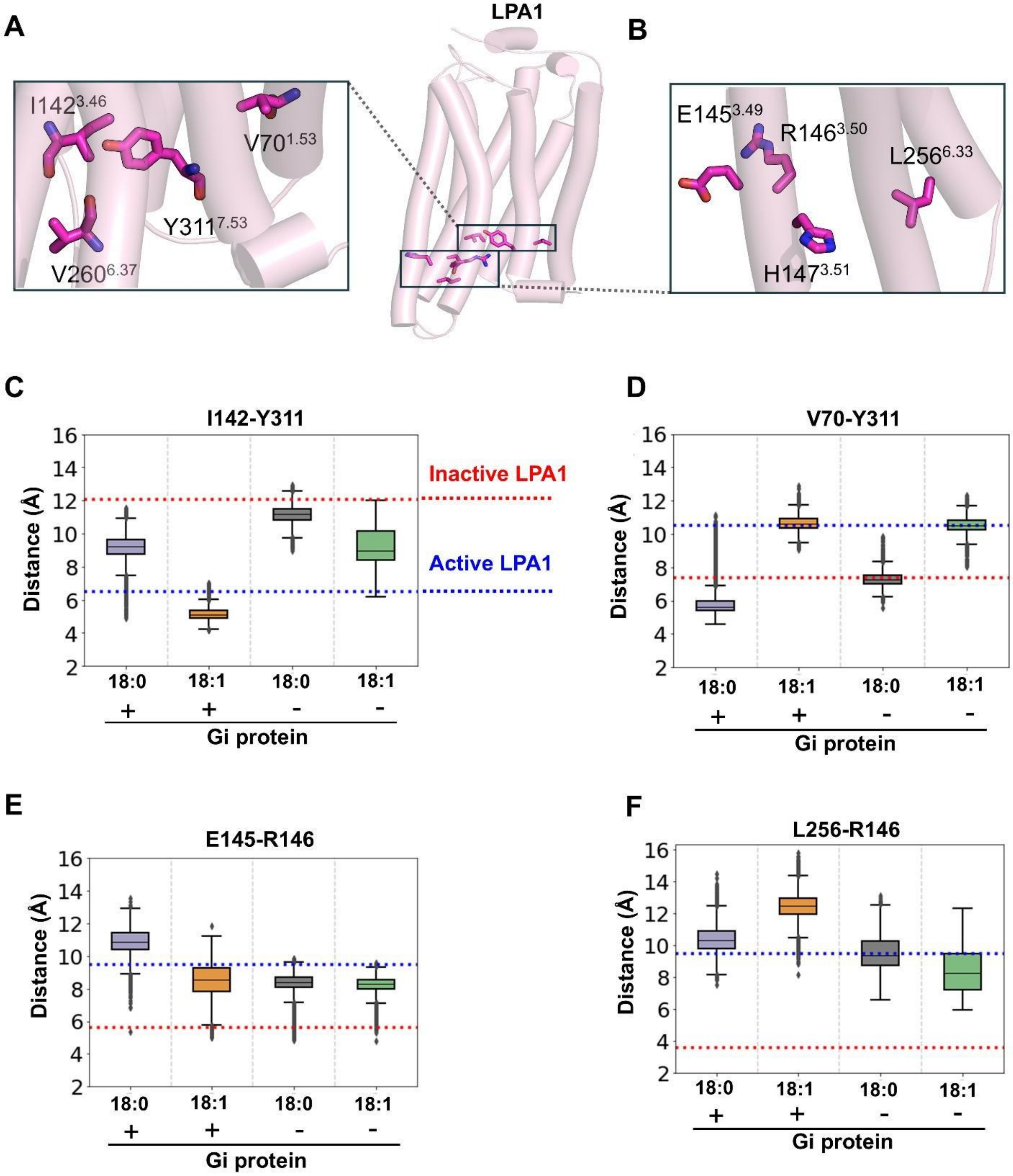


**Figure E. Comparative analyses of activation signatures in the LPA1 receptor in the different systems. A-B)** The cartoon representation of LPA1 with two activation switches highlighted in boxes. **C-F)** The distances between various residues present in the activation motifs in the presence of 18:0- and 18:1-LPA are compared with that of active (blue dotted line) and inactive state (red dotted line) receptors.

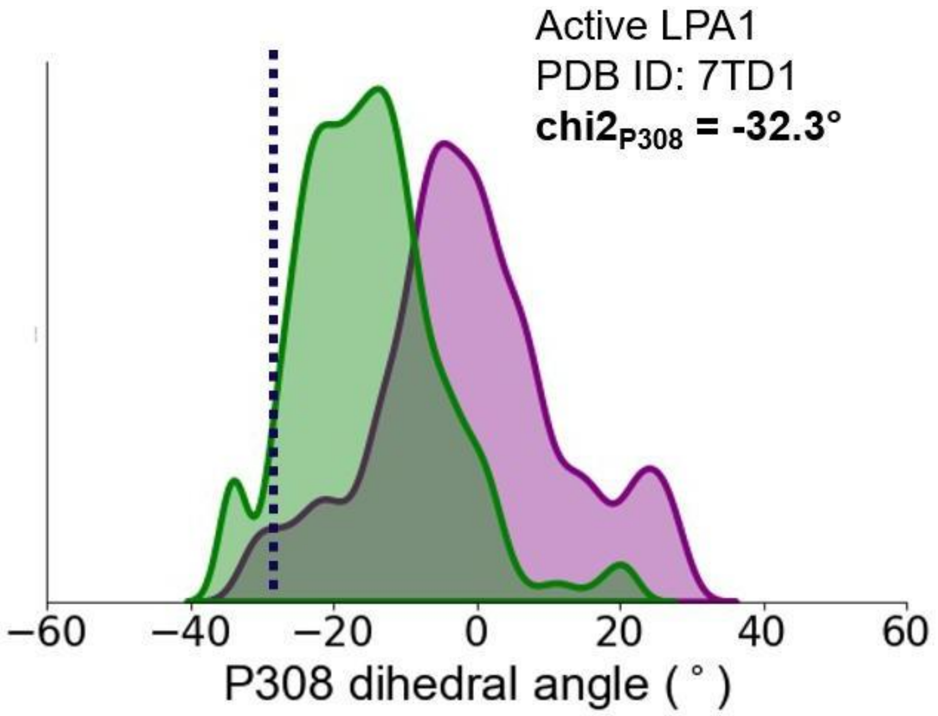


**Figure F. The chi2 dihedral angle of P308.** The density plot shows the rotameric changes in P308, part of one of the important activation switches, NPxxY, of LPA1 in the presence of 18:0 LPA (green) and 18:1 LPA ligands. The dotted vertical line shows the corresponding value (also given as text) observed in the active state LPA1 (PDB ID 7TD1).

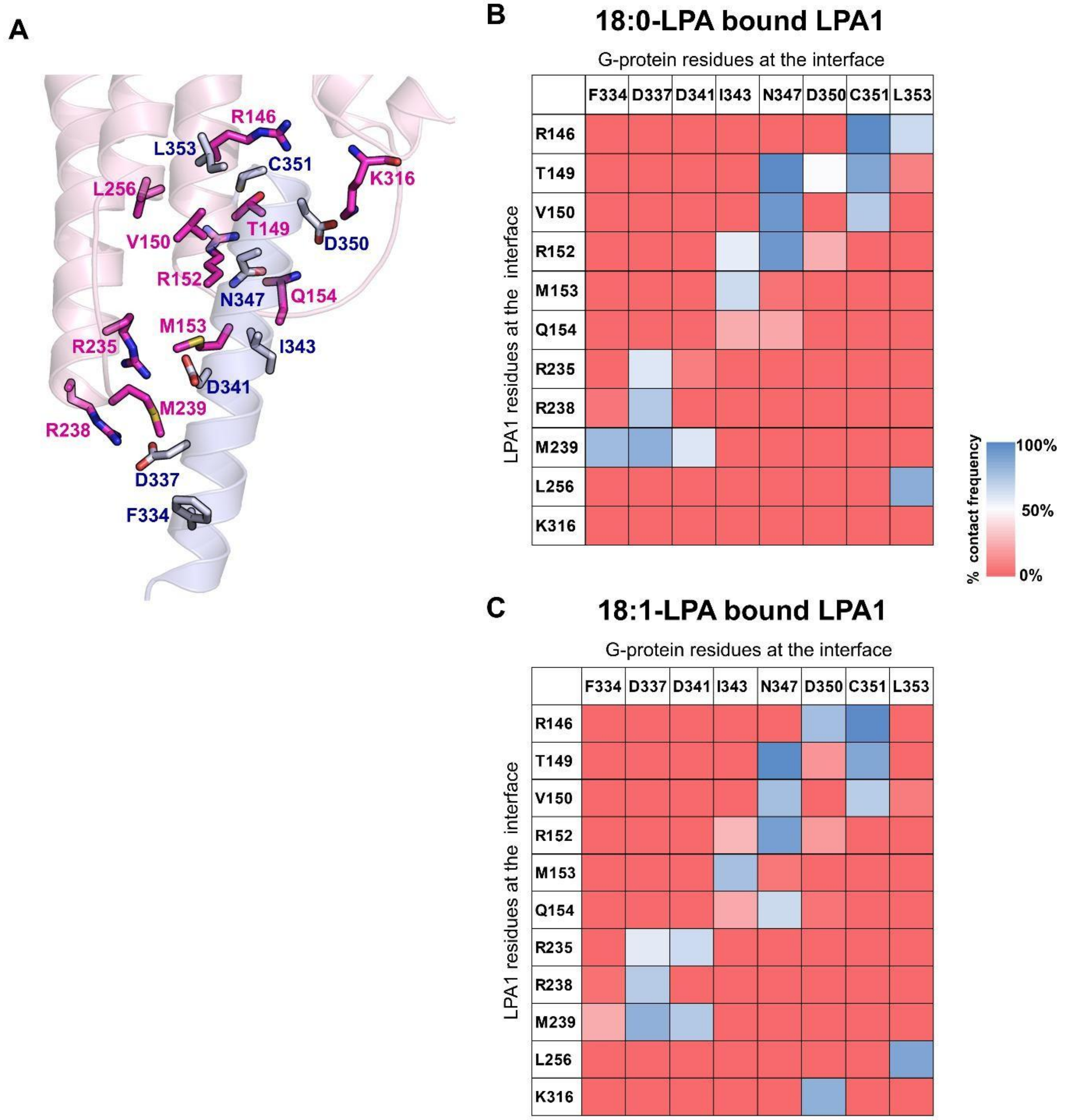


**Figure G. Residue-residue contacts at the LPA1 receptor and Gi protein coupling interface.** (A) Critical residue interactions at the LPA1-Gi protein interface, in the presence of two LPA species, were monitored as contact frequency during 500 ns MD simulations. LPA1 and Gi protein are shown in cartoon representations in pink and light blue; critical residues of the respective interacting species are shown in licorice representation in magenta and blue, respectively. (B and C) the heatmaps depict contact frequencies between critical residues at the interface in the presence of 18:0-LPA and 18:1-LPA, respectively.

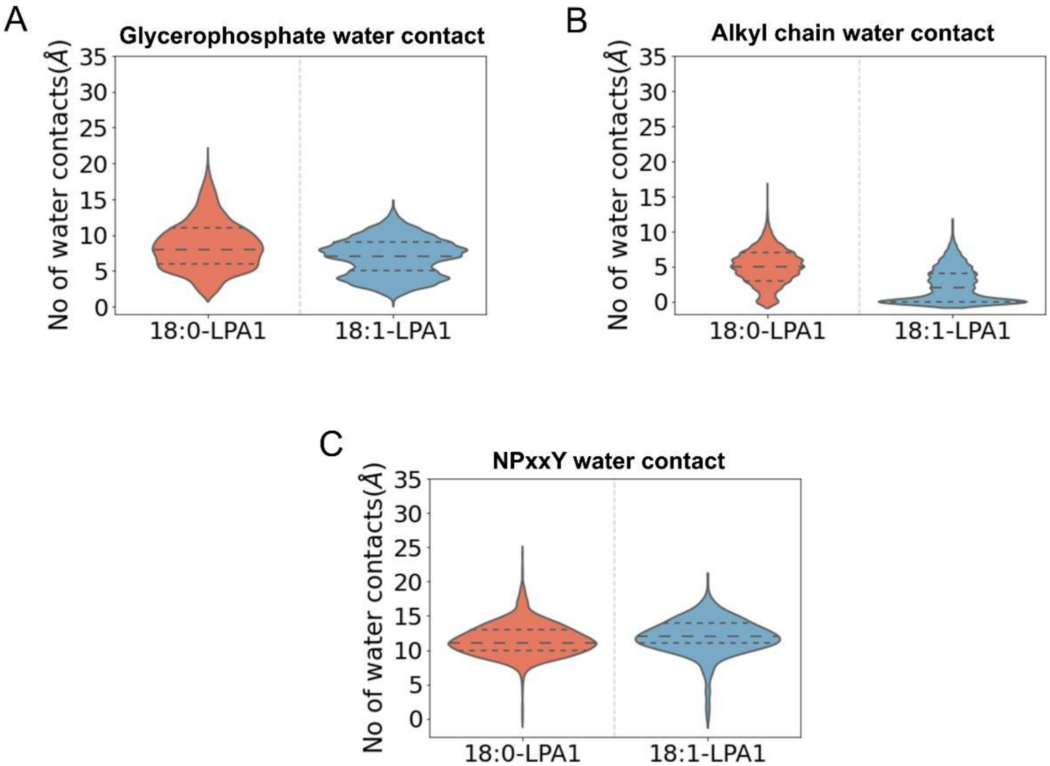


**Figure H. Water contact analyses of LPA species with LPA1**. (A-B)The number of water contacts with the glycerophosphate (A) and alkyl chain (B) components of 18:0-LPA and 18:1-LPA. (C) Water contacts with the conserved $N^{7.49}P^{7.50}xxY^{7.53}$ structural motif of the receptor.

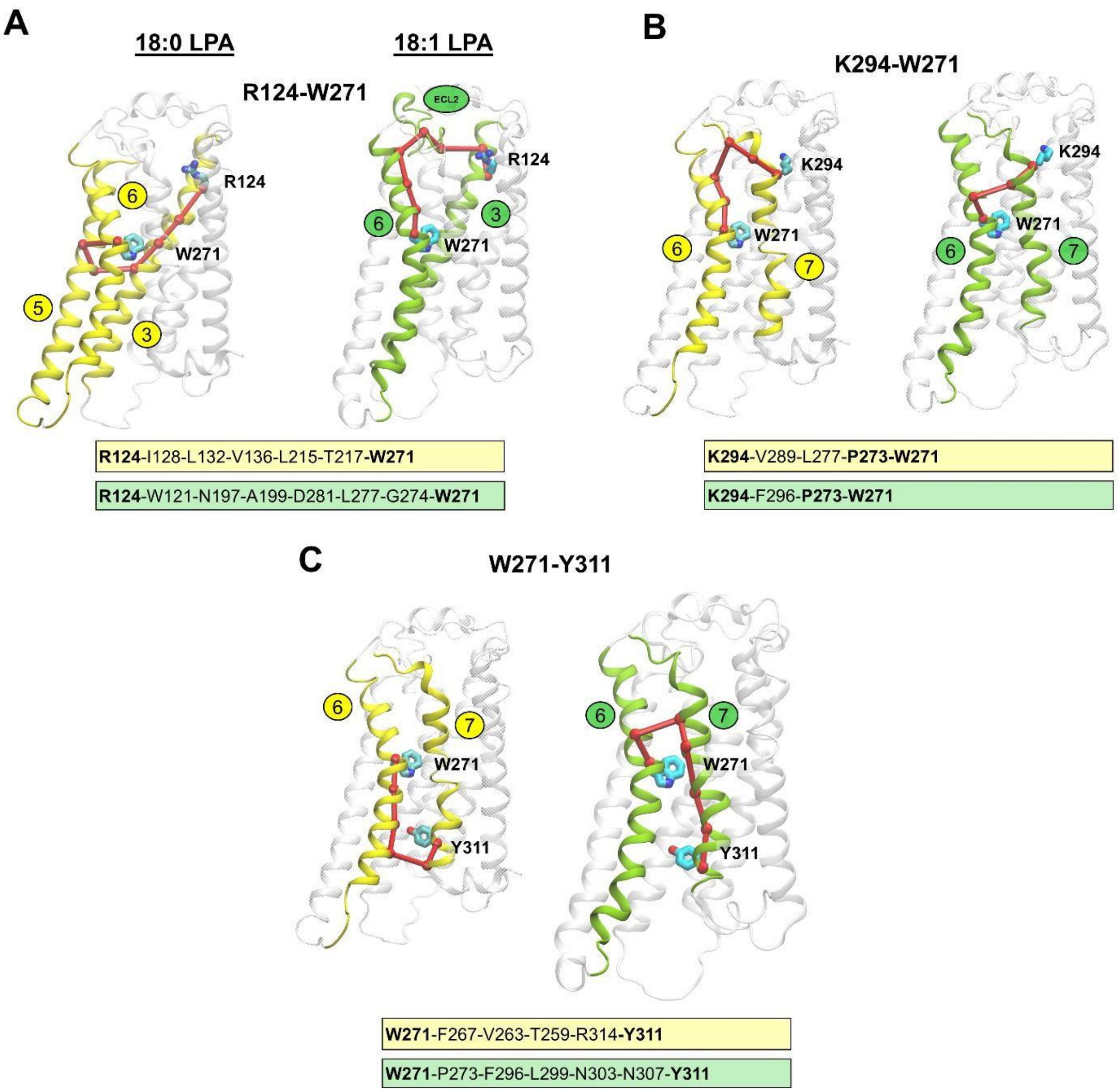


**Figure I. Residue-residue communication paths between residues at the orthosteric site and at activation switches**. In each case, 18:0-LPA- and 18:1-LPA-activated LPA1 receptors are depicted in cartoon representations, and participating helices are colored and labeled in yellow and green, respectively. A) The optimal communication path between R124$^{3.28}$ (binding site) and W271 (activation switch). B) The communication path between K294$^{7.36}$ and W271. (C) The communication path between two activation switches W271$^{6.48}$ (CWxP) and Y311$^{7.53,}$ from the NPxxY motif.

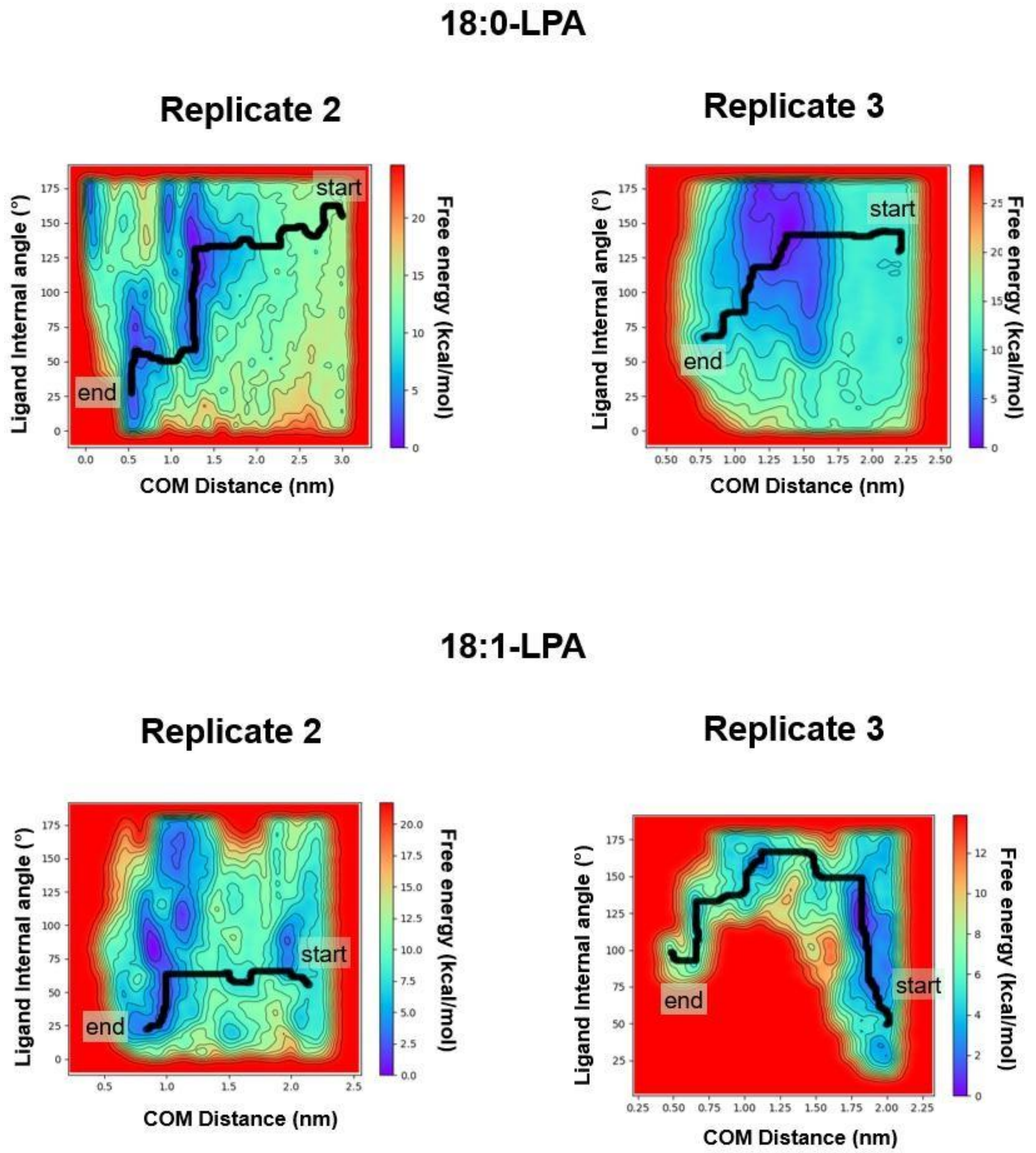


**Figure J. FES replicates 2 and 3 for 18:0-LPA and 18:1-LPA association with LPA1.** The 2D free-energy surfaces from independent replicates 2 and 3 of the WT-MetaD association simulations are shown for 18:0-LPA (top row) and 18:1-LPA (bottom row), using the same collective variables as in Figures 8 and 9 of the main text: the ligand-site COM distance (x-axis) and α (y-axis). The minimum energy path is shown as a bold black line and the starting (start) and final bound position (end) are labeled. The geometric consistency of the terminal state across all replicates for both ligands confirms the reproducibility of the reported entry mechanisms.

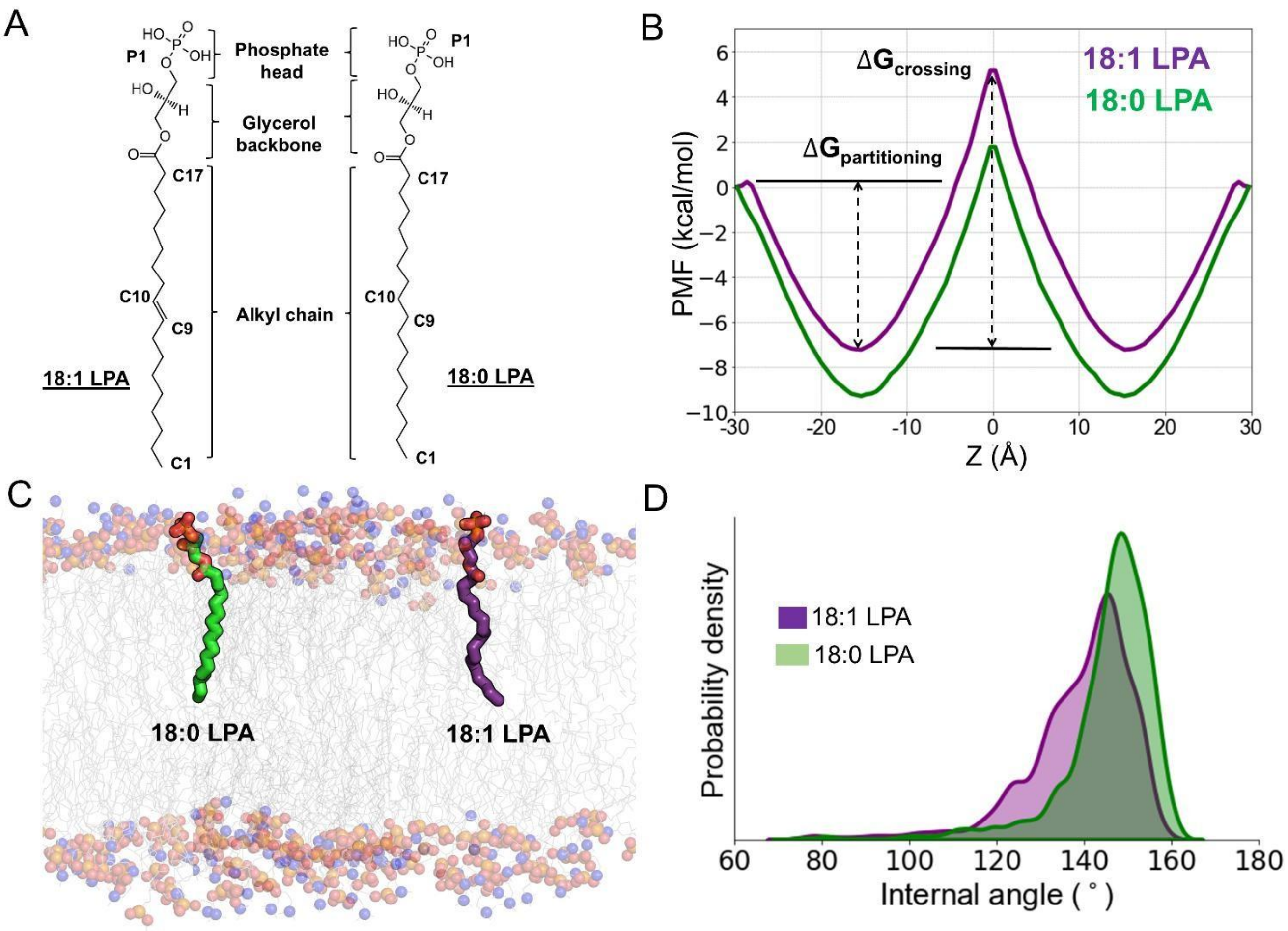


**Figure K. Membrane partitioning characteristics of 18:0 and 18:1-LPA species** (A) 2D-structures of the studied LPAs with their different functional groups and parts labeled. (B) The potential of mean force (PMF) curves for the ligands depicting the solvation-free energies for membrane partitioning and crossing the bilayer and energetically favorable bilayer locations. (C) The time-average preferred orientations of the ligands (in licorice representation) within the membrane. The membrane lipid head group atoms are depicted as balls: choline nitrogen (blue), glyceryl oxygen (red), and phosphorus of the phosphate group (olive green). The alkyl tails of the lipids are represented as lines (gray). (D) The probability density plot shows the internal angle (α) of the ligands (as defined in the Results section).

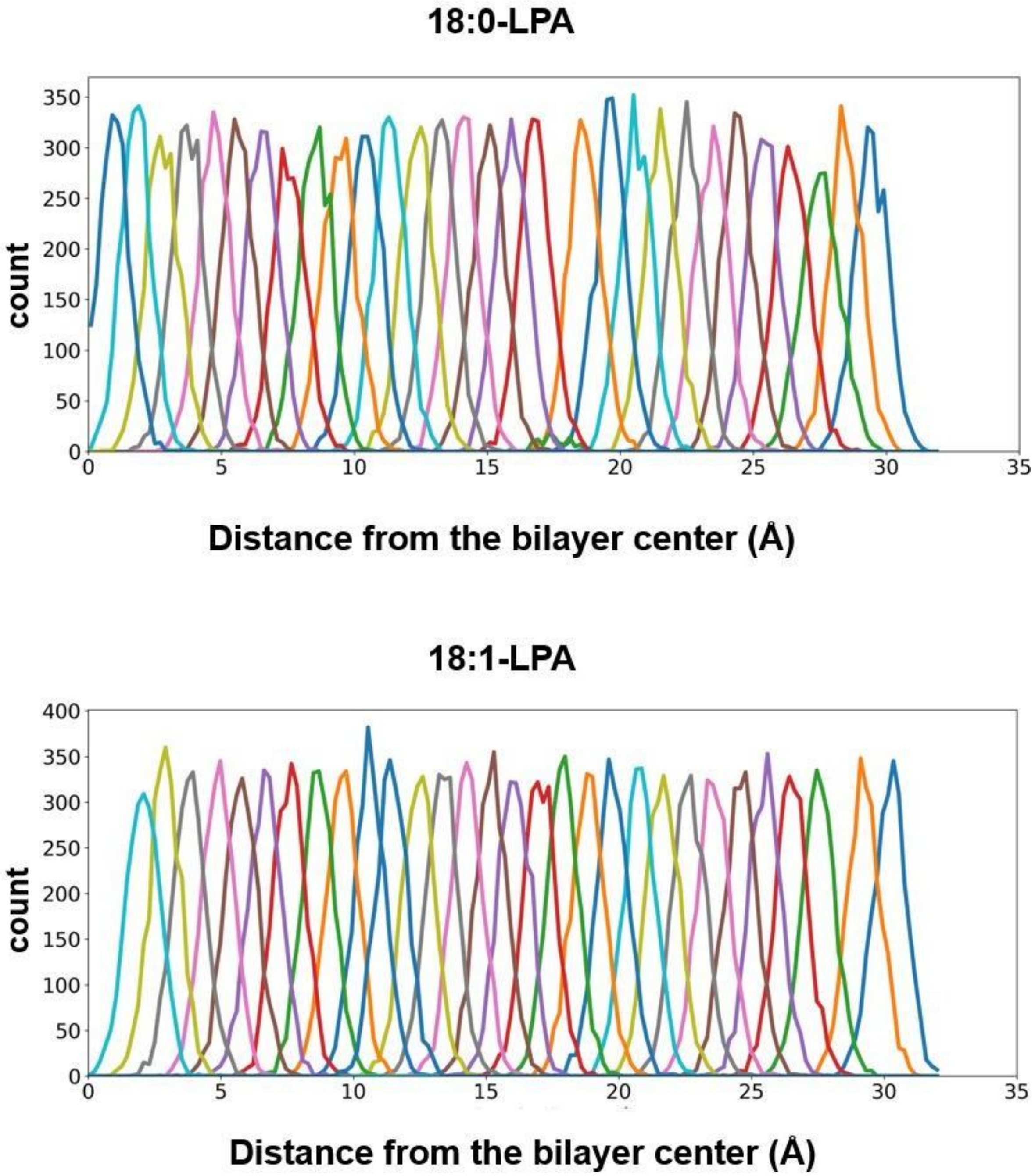


**Figure L.** Umbrella sampling histogram overlap for membrane partitioning simulations. Probability distributions of the ligand center-of-mass position along the bilayer normal (z-axis) for each umbrella sampling window for 18:0-LPA (top) and 18:1- LPA (bottom). Smooth and consistent overlap between adjacent windows confirms adequate phase-space sampling and the absence of gaps along the permeation coordinate.

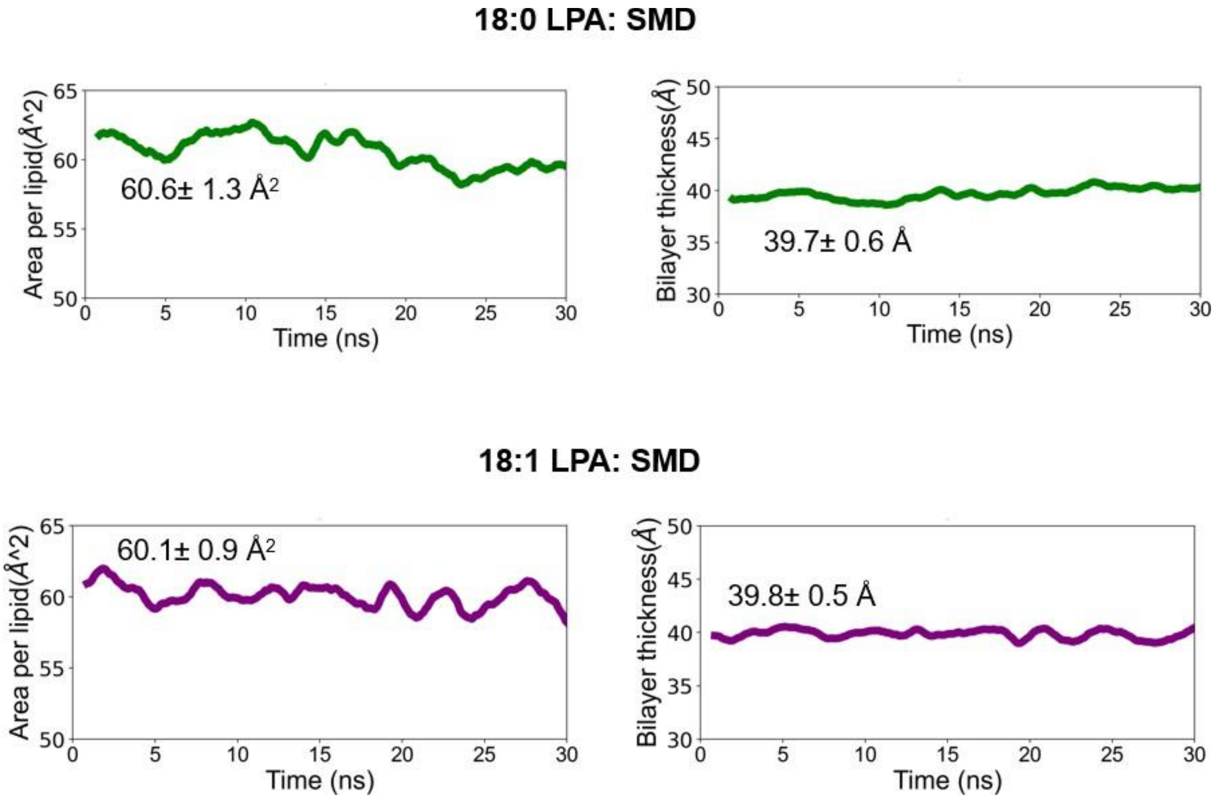


**Figure M. Bilayer structural integrity during SMD simulations.** Area per lipid (Å²) and bilayer thickness (Å) monitored throughout the SMD pulling simulations for 18:0-LPA (top) and 18:1-LPA (bottom). Values remain stable within ±2% of equilibrated reference values, confirming that ligand pulling did not perturb the membrane model.

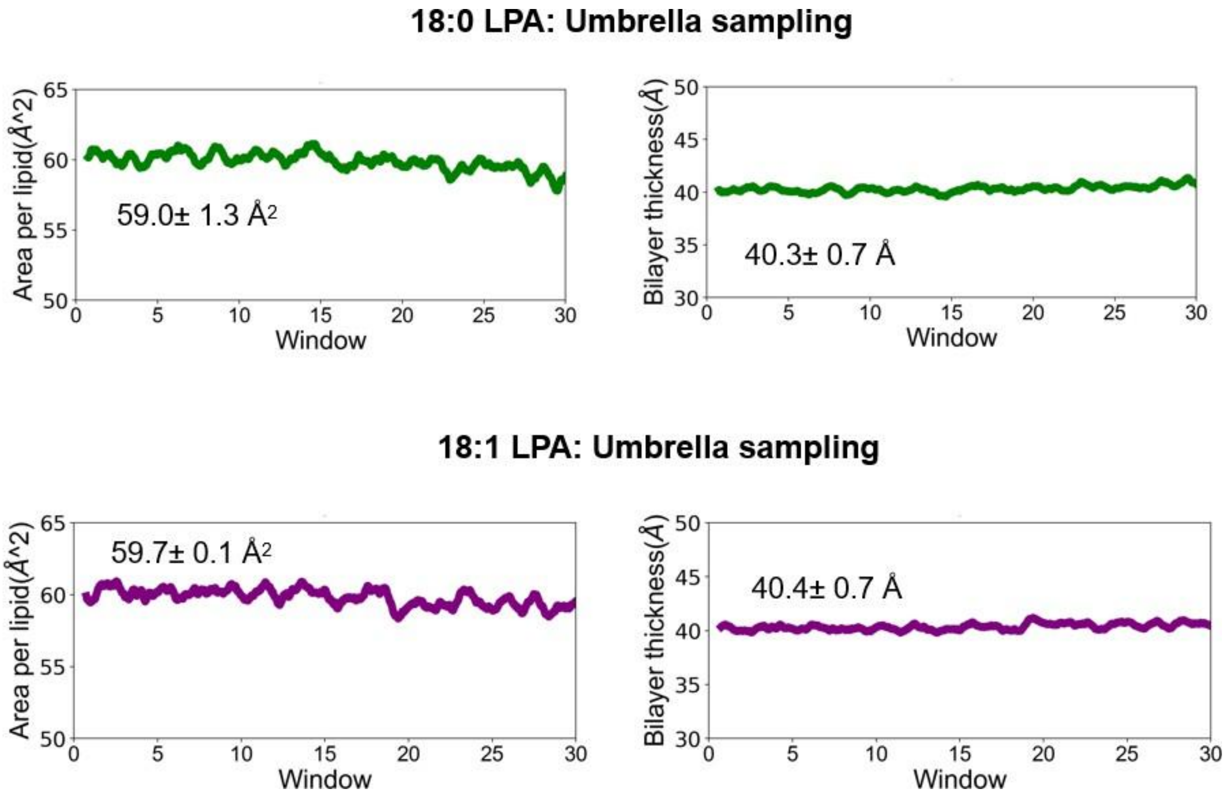


**Figure N. Bilayer structural integrity during umbrella sampling simulations.** Area per lipid (Å²) and bilayer thickness (Å) monitored throughout the umbrella sampling production runs for 18:0-LPA (left) and 18:1-LPA (right). Values remain stable within ±2% of equilibrated reference values across all windows, confirming membrane integrity throughout the PMF calculation.

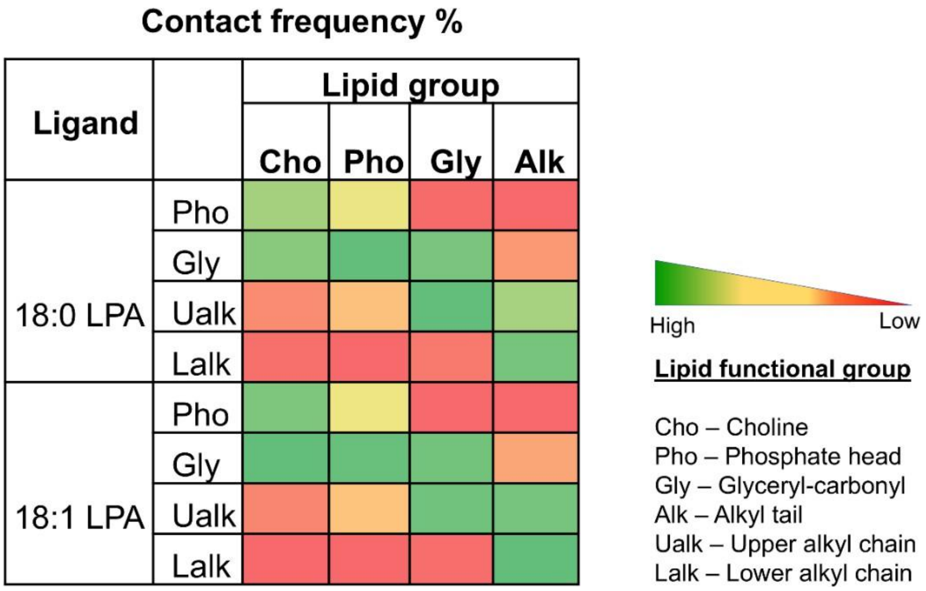


**Figure O. Ligand membrane contact analyses at the lowest energy windows from membrane partitioning simulations**. The contact frequency (%) of the ligand parts with various functional groups of the membrane lipids using a cut-off distance of 4 Å.

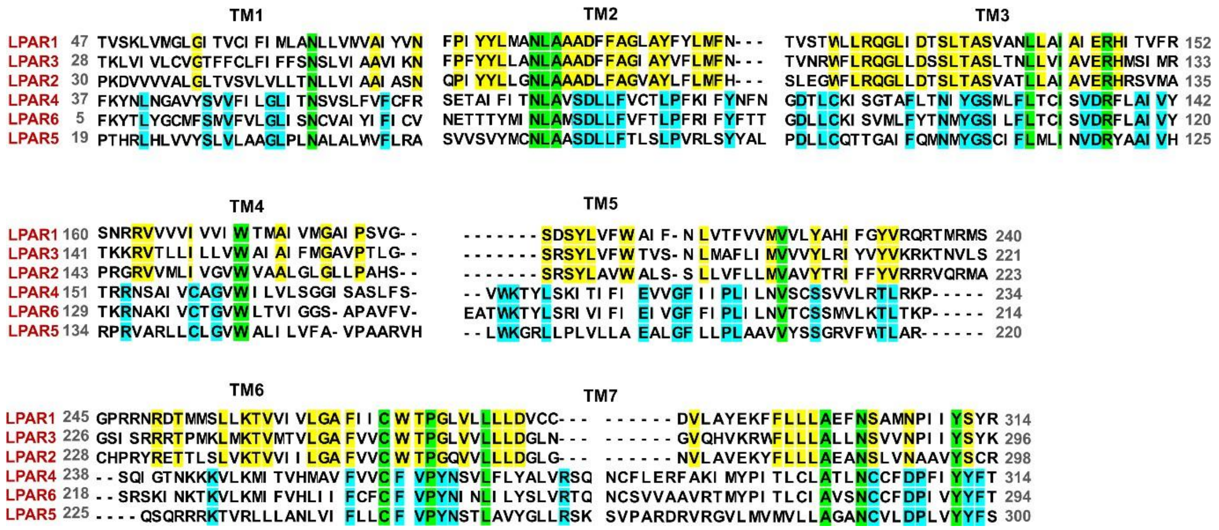

**Figure P. Multiple sequence alignment of the transmembrane region of lysophosphatidic acid receptor subtypes**. Residues conserved across all six subtypes are colored green, residues conserved among the endothelial differentiation gene (EDG) class are colored yellow, while the non-EDG conserved residues are colored cyan.
